# Integrative analysis of miRNA and mRNA profiles reveals that gga-miR-106-5p inhibits adipogenesis by targeting the *KLF15* gene in chickens

**DOI:** 10.1186/s40104-022-00727-x

**Published:** 2022-07-06

**Authors:** Weihua Tian, Xin Hao, Ruixue Nie, Yao Ling, Bo Zhang, Hao Zhang, Changxin Wu

**Affiliations:** 1 National Engineering Laboratory for Animal Breeding, Beijing Key Laboratory for Animal Genetic Improvement, College of Animal Science and Technology, China Agricultural University, Beijing, 100193 China; 2 Sanya Institute of China Agricultural University, Hainan 572025 Sanya, China

**Keywords:** Abdominal fat, Adipogenesis, Chickens, gga-miR-106-5p, KLF15, MiRNA

## Abstract

**Background:**

Excessive abdominal fat deposition in commercial broilers presents an obstacle to profitable meat quality, feed utilization, and reproduction. Abdominal fat deposition depends on the proliferation of preadipocytes and their maturation into adipocytes, which involves a cascade of regulatory molecules. Accumulating evidence has shown that microRNAs (miRNAs) serve as post-transcriptional regulators of adipogenic differentiation in mammals. However, the miRNA-mediated molecular mechanisms underlying abdominal fat deposition in chickens are still poorly understood. This study aimed to investigate the biological functions and regulatory mechanism of miRNAs in chicken abdominal adipogenesis.

**Results:**

We established a chicken model of abdominal adipocyte differentiation and analyzed miRNA and mRNA expression in abdominal adipocytes at different stages of differentiation (0, 12, 48, 72, and 120 h). A total of 217 differentially expressed miRNAs (DE-miRNAs) and 3520 differentially expressed genes were identified. Target prediction of DE-miRNAs and functional enrichment analysis revealed that the differentially expressed targets were significantly enriched in lipid metabolism-related signaling pathways, including the PPAR signaling and MAPK signaling pathways. A candidate miRNA, gga-miR-106-5p, exhibited decreased expression during the proliferation and differentiation of abdominal preadipocytes and was downregulated in the abdominal adipose tissues of fat chickens compared to that of lean chickens. gga-miR-106-5p was found to inhibit the proliferation and adipogenic differentiation of chicken abdominal preadipocytes. A dual-luciferase reporter assay suggested that the *KLF15* gene, which encodes a transcriptional factor, is a direct target of gga-miR-106-5p. gga-miR-106-5p suppressed the post-transcriptional activity of *KLF15*, which is an activator of abdominal preadipocyte proliferation and differentiation, as determined with gain- and loss-of-function experiments.

**Conclusions:**

gga-miR-106-5p functions as an inhibitor of abdominal adipogenesis by targeting the *KLF15* gene in chickens. These findings not only improve our understanding of the specific functions of miRNAs in avian adipogenesis but also provide potential targets for the genetic improvement of excessive abdominal fat deposition in poultry.

**Supplementary Information:**

The online version contains supplementary material available at 10.1186/s40104-022-00727-x.

## Background

In recent decades, intensive genetic selection in broilers has contributed to rapid growth rates and higher meat yields in chicken, but this has been accompanied by the excessive deposition of body fat, particularly abdominal fat [1]. Abdominal fat is an important carcass trait in chickens. However, excessive abdominal fat is undesirable because it represents wasteful dietary energy input, inefficient meat production, and negative reproductive performance. Higher abdominal fat also enhances the nitrogen and phosphate contents of excrement and results in serious pollution to the environment [2, 3]. Therefore, increasing concerns over excessive abdominal fat have attracted the attention of researchers. Abdominal fat weight and percentage are major phenotypic indices that directly reflect body fat deposition. These traits have high heritability and show positive genetic correlation with each other, but cannot be determined directly in vivo. Accordingly, these are not suitable traits for breeding improvement via traditional selection methods to reduce abdominal fat deposition [4–6]. Therefore, an in-depth study of the molecular regulatory mechanisms underlying abdominal fat deposition is of great significance, as it would facilitate the genetic improvement of abdominal fat deposition.

Abdominal fat expands via increasing the number of preadipocytes (proliferation) and promoting the differentiation of preadipocytes into mature adipocytes (hypertrophy) [7]. Preadipocytes mainly proliferate during embryonic development and at an early life stage after birth, and hypertrophy primarily depends on lipid droplet accumulation in mature adipocytes during animal growth. The proliferation and differentiation of preadipocytes are well-orchestrated multistep processes that involve the sequential activation of numerous transcription factors, such as the CCAAT/enhancer-binding protein (C/EBP) family, peroxisome proliferator activated receptor γ (PPARγ), Wnt, Kruppel-like factors (KLFs), unigenes closely associated with lipid metabolism, and endocrine factors [8–11].

Given the complexity of regulatory mechanisms underlying adipogenesis, it is likely that noncoding RNAs, such as microRNAs (miRNAs), are operative in this process. These miRNAs may be used as novel biomarkers for relieving abdominal fat accumulation in chickens, and their identification may allow the development of novel therapeutic targets for obesity in humans [12]. miRNAs are a subclass of endogenous noncoding RNAs that are approximately 18–25 nucleotides (nt) in length. They function as important regulators that post-transcriptionally trigger the silencing of target mRNAs via their cleavage or translation inhibition by perfectly or imperfectly binding to the 3′ untranslated regions (3′ UTRs) of mRNAs [13, 14]. Increasing evidence has revealed that miRNAs are involved in adipogenesis in mammals [15–17]. In chickens, although many miRNAs and miRNA-mRNA regulatory networks have been identified in abdominal adipose tissues, their specific effects on adipogenesis are poorly understood. To date, only a few miRNAs have been validated as functional regulators in the development of abdominal adipose tissues in chickens. Of these, gga-miR-19b-3p may accelerate the proliferation of abdominal preadipocytes and their subsequent differentiation into mature adipocytes in chickens by inhibiting its target gene, acyl-CoA synthetase long-chain family member 1 (*ACSL1*), which drives the catalysis of long-chain fatty acids into acyl-CoA (a source of lipid synthesis) [18]. Moreover, the overexpression of gga-miR-206 may result in the inhibition of adipogenesis via the downregulation of its target gene, Kruppel-like factor 4 (*KLF4*), which is an important activator of adipogenesis in chickens [19]. gga-miR-21 may inhibit abdominal preadipocyte proliferation in chickens, in part by downregulating the mRNA and protein levels of the Kruppel-like factor 5 (*KLF5*) gene [20]. Moreover, the miR-17-92 cluster is known to promote abdominal preadipocyte proliferation in chickens [21].

This study aimed to investigate the expression profiles of miRNAs, mRNAs, and transcription factors in the abdominal adipocytes of chickens at different stages of differentiation (0, 12, 48, 72, and 120 h) and evaluate the differential expression, regulatory network profiles, and functions of the expressed miRNAs and mRNAs. Based on the obtained information, we screened a candidate miRNA (gga-miR-106-5p) and a potential target gene (Kruppel-like factor 15 [*KLF15*]) involved in abdominal adipocyte differentiation in chickens. *KLF15* encodes a key regulatory transcriptional factor involved in adipogenesis; the *KLF15* gene was identified as a direct target of gga-miR-106-5p using a dual-luciferase reporter assay. The dynamic expression profiles of gga-miR-106-5p and *KLF15* were detected in vivo and in vitro. Effects of the gga-miR-106-5p and *KLF15* gene on the proliferation and differentiation of chicken abdominal preadipocytes were further validated. Our study suggests that gga-miR-106-5p plays a crucial role in regulating adipogenesis by silencing *KLF15* mRNA expression in chickens, and these findings enrich our understanding of the molecular genetic controls underlying abdominal fat deposition in poultry.

## Methods

### Experimental animals and sample preparation

A populations of female Arbor Acres (AA) broilers were raised under the same conditions and were humanely slaughtered at 42 days old. According to abdominal fat percentage (AbFP) measured, eight broilers with extreme high AbFP (HAbF, 1.83 ± 0.23%) and eight with extreme low AbFP (LAbF, 0.43 ± 0.24%) were selected to collected abdominal fat tissues. Then, a portion of the abdominal adipose tissue samples was immediately snap frozen in liquid nitrogen and stored at − 80 °C for RNA extraction. The remaining samples were soaked in 4% paraformaldehyde (Solarbio, Beijing, China) and used to prepare paraffin sections for hematoxylin and eosin (HE) staining.

### Cell culture

Immortalized chicken preadipocytes 2 (ICP2) cell lines were established by infecting recombinant retroviruses expressing chicken telomerase reverse transcriptase and telomerase RNA into primary chicken preadipocytes isolated from the abdominal adipose tissues of 10-day-old AA broilers [22]. The ICP2 cells were obtained from the Key Laboratory of Chicken Genetics and Breeding, Ministry of Agriculture (Northeast Agricultural University) and maintained in a basal medium consisting of Dulbecco’s modified Eagle’s medium F12 (DMEM-F12) (Gibco, Gaithersburg, MD, USA) supplemented with 10% fetal bovine serum (FBS) (Gibco) and 1% penicillin-streptomycin (Gibco). Human embryonic kidney 293 T cells and chicken embryonic fibroblast DF-1 cells were obtained from the American Type Culture Collection (ATCC, Manassas, VA, USA). The 293 T cells and DF-1 cells were maintained in DMEM (Gibco) supplemented with 10% FBS (Gibco) and 1% penicillin-streptomycin (Gibco). All cells were cultured at 37 °C and 5% CO_2_ in a humidified incubator.

### Adipogenic differentiation assay in chicken preadipocytes

While growing to 80–90% confluence, ICP2 cells were cultured in 6-well plates at an adjusted density of 1 × 10^5^ cells/mL. The cells were divided into eight groups (with six biological replicates per group) and treated with a differentiation medium containing basal DMEM-F12 medium. To induce adipogenic differentiation, the basal medium was supplemented with 160 μmol/L sodium oleate (Sigma, St. Louis, MO, USA) dissolved in sterile deionized water. The differentiation medium was changed daily. The cells were washed thrice in phosphate-buffered saline (PBS) (Gibco) and harvested at 0, 6, 12, 24, 48, 72, 96, and 120 h post-differentiation. The samples were stored at − 80 °C until RNA extraction.

### High-throughput sequencing of RNA (RNA-seq) library construction and sequencing

To investigate the dynamic expression profiles of miRNAs and mRNAs in the developing adipocytes, we collected differentiated adipocytes at 0, 12, 48, 72, and 120 h (three biological replicates per group) and used these for RNA-seq. Total RNA was extracted using TRIzol reagent (Ambion, Austin, TX, USA). RNA concentration and integrity were measured using a NanoDrop 2000 Spectrophotometer (Thermo Fisher Scientific, Wilmington, DE, USA) and an Agilent 2100 Bioanalyzer (Agilent Technologies, Santa Clara, CA, USA). RNA samples with a 28S/18S band intensity ratio of > 1.5, RNA integrity of 8.0–10.0, OD_260/280 nm_ of 1.8–2.0, and OD_260/230 nm_ of 2.0–2.3 were used for RNA-seq.

For miRNA profiling, we prepared small RNA libraries using 3 μg of RNA from each library and the NEBNext® Multiplex Small RNA Library Prep Set for Illumina® (New England Biolabs, Ipswich, MA, USA) according to the manufacturer’s instructions. For mRNA profiling, we prepared Ribo-Zero RNA-seq libraries using 3 μg of RNA from each library and the Ribo-Zero rRNA Removal Kit (Epicenter, Madison, WI, USA) and NEBNextR Ultra™ Directional RNA Library Prep Kit for Illumina® (New England Biolabs) according to the manufacturer’s recommendations. All the generated RNA-seq data have been deposited in the National Center for Biotechnology Information (NCBI) Sequence Read Archive (SRA) database under accession number PRJNA732104, and are included in our published article [23].

### Sequence data processing

For miRNA-seq, raw reads were cleaned using in-house Perl scripts after removing the adaptor sequences, low-quality reads, sequences with over 10% poly-N, and reads shorter than 18 nt or longer than 30 nt. The clean reads were matched with the Silva, GtRNAdb, Rfam, and Repbase databases to filter out ribosomal RNA (rRNA), transfer RNA (tRNA), small nuclear RNA (snRNA), small nucleolar RNA (snoRNA), and repeat-associated RNA. Finally, we obtained unannotated reads containing miRNAs. The unannotated reads were aligned to the reference chicken genome (Gallus_gallus-6.0) using the Bowtie software [24]. To identify the known miRNAs, the mapped reads were aligned to known mature miRNAs from miRbase [25], with the maximum number of mismatched bases set to 1. We also used miRDeep2 to predict novel miRNAs. miRNA expression was normalized to transcripts per million, and differential analysis of miRNA expression was performed using DESeq2 [26]. Differentially expressed miRNAs (DE-miRNAs) were identified as those with |log_2_ fold change| ≥0.585 and a *P*-value of < 0.05. The targets of miRNAs were obtained from the intersection of miRanda [27] and TargetScan [28]. The clustering of short time-series miRNA expression was analyzed and visualized with the Short Time-series Expression Miner (STEM) software [29].

For mRNA-seq, the adapters, low-quality reads, and reads with over 10% poly-N were removed from raw reads to yield clean reads. The clean reads were aligned to the Galgal 6 chicken reference genome to generate mapped reads using HiSAT2 [30]. We then assembled the mapped reads into transcripts and quantified gene expression (normalized by fragments per kilobase of transcript per million fragments mapped) using the StringTie software [31]. DESeq2 was used to analyze differentially expressed genes (DEGs); genes with |log_2_ fold change| ≥ 0.585 and false discovery rate ≤ 0.05 were identified as DEGs. Gene Ontology and Kyoto Encyclopedia of Genes and Genomes pathway enrichment analysis of DEGs and differentially expressed targets of DE-miRNAs were performed using the R package clusterProfiler [32]. Transcriptional factors in chickens were projected and classified using AnimalTFDB [33]. The promoter sequences 2000 bp upstream from the transcription start site (TSS) were used to search for the KLF15 motif with a *P-*value of ≤ 0.0001 using the online software FIMO [34]. The vertebrate KLF15 matrices (MA1513.1) were obtained from the online software JASPAR [35].

### Complementary DNA (cDNA) synthesis and quantitative real-time PCR (qRT-PCR)

To validate the miRNA expression data, 2 μg of total RNA from each sample was reverse transcribed into cDNA using the miRcute Plus miRNA First-Strand cDNA Kit (TIANGEN, Beijing, China) following the manufacturer’s recommendations. We performed SYBR green-based qRT-PCR in triplicate on a BioRad CFX96 Real Time PCR system (BioRad, USA). The 20 μL reaction volume contained 10 μL 2× miRcute Plus miRNA PreMix (SYBR&ROX) (TIANGEN), 8.2 μL RNase-free water, 0.4 μL each of forward and reverse primers (10 μmol/L), and 1 μL cDNA (approximately 300 ng). The qRT-PCR amplification protocol consisted of initial denaturation at 95 °C for 15 min, 40 cycles of denaturation at 94 °C for 20 s, annealing at 60 °C for 30 s, and extension at 72 °C for 34 s, and a final melting/dissociation curve stage. The housekeeping gene *U6* served as an internal control to normalize the relative miRNA expression.

To verify the mRNA expression data, 2 μg total RNA from each sample was reverse transcribed into cDNA using the FastKing RT Kit (with gDNase) (TIANGEN) following the manufacturer’s instructions. We performed SYBR green-based qRT-PCR in triplicate on a BioRad CFX96 Real Time PCR system (BioRad). The 20 μL reaction volume contained 10 μL 2× Talent qPCR PreMix (SYBR Green) (TIANGEN), 7.8 μL RNase-free water, 0.6 μL each of forward and reverse primers (10 μmol/L), and 1 μL cDNA (approximately 300 ng). The qRT-PCR amplification protocol consisted of initial denaturation at 95 °C for 3 min, 40 cycles of denaturation at 95 °C for 5 s, annealing at optimum temperature for 10 s, and extension at 72 °C for 15 s, and a final melting/dissociation curve stage. The housekeeping gene *GAPDH* served as an internal control to normalize the relative mRNA expression.

The relative expression levels of miRNAs and mRNAs were calculated using the 2^−∆∆Ct^ method. The qRT-PCR primers used to quantify miRNA expression were designed using the miRprimer2 software. The qRT-PCR primers used to quantify mRNA expression were designed using NCBI Primer-BLAST [36]. All primers were synthesized by SinoGenoMax (Beijing, China) (Additional File 1: Table S1).

### HE staining

The chicken abdominal fat tissue was immobilized in 4% paraformaldehyde for 30 min and embedded in paraffin to prepare paraffin sections. The sections were stained with hematoxylin for 15 min. Following differentiation in 1% hydrochloric acid-ethanol for several seconds, the paraffin sections were subsequently stained with 0.5% eosin for 3 min, followed by gradient alcohol dehydration for 2 min and vitrification with dimethylbenzene for 2 min. Cell nuclei were stained blue, and the cytoplasm was stained pink. The cells were microscopically observed and photographed using an Echo Revolve microscope (Echo Laboratories, San Diego, CA, USA).

### Oil red O staining

The ICP2 cells were washed thrice with PBS and fixed in 4% formaldehyde for 30 min. After washing thrice again with PBS, the cells were stained with Oil Red O (Sigma, St. Louis, MO, USA) dissolved in 100% isopropyl alcohol for 30 min. Following infiltration with 60% isopropyl alcohol for 10 s, the cells were washed thrice with PBS and imaged under a microscope. Next, intracellular Oil Red O was dissolved by infiltration with 100% isopropyl alcohol for 5 min, and we evaluated the content of lipid droplets via spectrophotometrically measuring the absorbance at 490 nm.

### Nile red fluorescent staining

The cells were washed thrice with PBS, fixed in 4% formaldehyde for 10 min, again washed thrice with PBS for 10 min, and incubated with Nile red fluorescent dye (Applygen Technologies Inc., Beijing, China) for 10 min at 25 °C. After washing with PBS, the cells were incubated with DAPI for 10 min to stain the nuclei at 25 °C. The cells were microscopically observed and photographed using an Echo Revolve fluorescence microscope (Echo Laboratories).

### Cell counting Kit-8 (CCK-8) assay

The cells were seeded in 96-well plates and cultured in a basal medium. At 12, 24, 48, 72, 96, and 120 h post-transfection, cell proliferation was monitored using the Cell Counting Kit-8 (Beyotime Biotechnology, Shanghai, China) according to the manufacturer’s protocol. After 1 h of incubation, absorbance at 450 nm was measured using a SpectraMax® i3x Multi-Mode Microplate Reader (Molecular Devices Corporation, Sunnyvale, CA, USA).

### 5-Ethynyl-2-deoxyuridine (EdU) assay

The cells were seeded in 12-well plates and cultured in basal medium. At 48 h post-transfection, the cells were stained for 2 h using a BeyoClick™ EdU Cell Proliferation Kit with Alexa Fluor 555 (Beyotime, Shanghai, China) following the manufacturer’s protocol. Cell nuclei were stained blue, and the EdU-positive cells were stained red. The cells were microscopically observed and photographed using an Echo Revolve fluorescence microscope (Echo Laboratories).

### Plasmid construction and RNA oligonucleotide synthesis

To determine whether gga-miR-106-5p targets the *KLF15* gene, the 3′ UTR region of the *KLF15* gene containing a putative gga-miR-106-5p binding site and an insert containing the *Xho*I and *Not*I restriction enzyme sites was amplified via PCR using PrimeSTAR Max Premix (Takara, Kyoto, Japan) according to the manufacturer’s specifications. The fragments were then cloned into the *Xho*I and *Not*I (Takara, Kyoto, Japan) double-digested psi-CHECK™-2 vector (Promega, Madison, WI, USA), using the Trelief™ SoSoo Cloning Kit (TSINGKE, Beijing, China), to construct the wild-type plasmid miR-106-5p-KLF15-WT. Similarly, the gga-miR-106-5p binding site to the *KLF15* gene was deleted from the 3′ UTR region, which was then amplified using overlap PCR and cloned to construct the mutant-type plasmid miR-106-5p-KLF15-Mut. All primers were synthesized by SinoGenoMax (Additional File 1: Table S1).

To construct an overexpression vector for the *KLF15* gene, the coding sequence of *KLF15* (with a deleted termination codon and inserted *Nhe*I and *Sac*II restriction enzyme sites) was PCR-amplified using PrimeSTAR® Max DNA Polymerase (Takara) and cDNA from chicken abdominal fat. This was cloned into the *Nhe*I and *Sac*II (Takara) double-digested pcDNA3.1-EGFP vector (Invitrogen, Carlsbad, CA, USA), using the Trelief™ SoSoo Cloning Kit (TSINGKE), to construct the pcDNA3.1-KLF15-EGFP plasmid. Plasmid DNA was extracted and purified using the EndoFree Maxi Plasmid Kit (TIANGEN) following the manufacturer’s instructions. All primers were synthesized by SinoGenoMax (Additional File 1: Table S1).

The siRNA oligonucleotides (sense: CUGAAUUCGCUGUGGAUAUTT; antisense: AUAUCCACAGCGAAUUCAGGC) siKLF15, designed to specifically knockdown the *KLF15* gene, and miR-106-5p agomir, which causes the overexpression of gga-miR-106-5p, as well as their corresponding negative controls (NC), were synthesized by GenePharma Co., Ltd. (Shanghai, China). All cell transfections were performed using Lipofectamine 3000 (Invitrogen) following the manufacturer’s protocol.

### Dual-luciferase reporter assay

To determine the interaction between miR-106-5p and its potential target gene (*KLF15*), 293 T cells and DF1 cells were seeded in 24-well plates and co-transfected in triplicate with 80 nmol/L miR-106-5p agomir or agomir NC and 500 ng of the aforementioned wild-type or mutant plasmids per well. At 48 h post-transfection, the cells were washed thrice with PBS (Gibco) and lysed with 1× passive lysis buffer (Promega, Madison, WI, USA) for 15 min. Firefly luciferase and *Renilla* luciferase signals were measured using the Dual-Luciferase® Reporter Assay System (Promega, Madison, WI, USA) on a SpectraMax® i3x Multi-Mode Microplate Reader (Molecular Devices Corporation, Sunnyvale, CA). The *Renilla* luciferase signal was normalized to the firefly luciferase signal. All reactions were performed in triplicate.

### Statistical analysis

All data are presented as mean ± standard deviation (SD). Statistically significant differences between two experimental groups were determined via the *t*-test using the SPSS 23.0 software (IBM, Chicago, IL, USA). Significance was set at *P value < 0.05, and extreme significance was set at **P value < 0.01. The results were illustrated using GraphPad Prism 8 (GraphPad Software, San Diego, CA, USA).

## Results

### Summary of small RNA sequencing

**Fig. 1:**
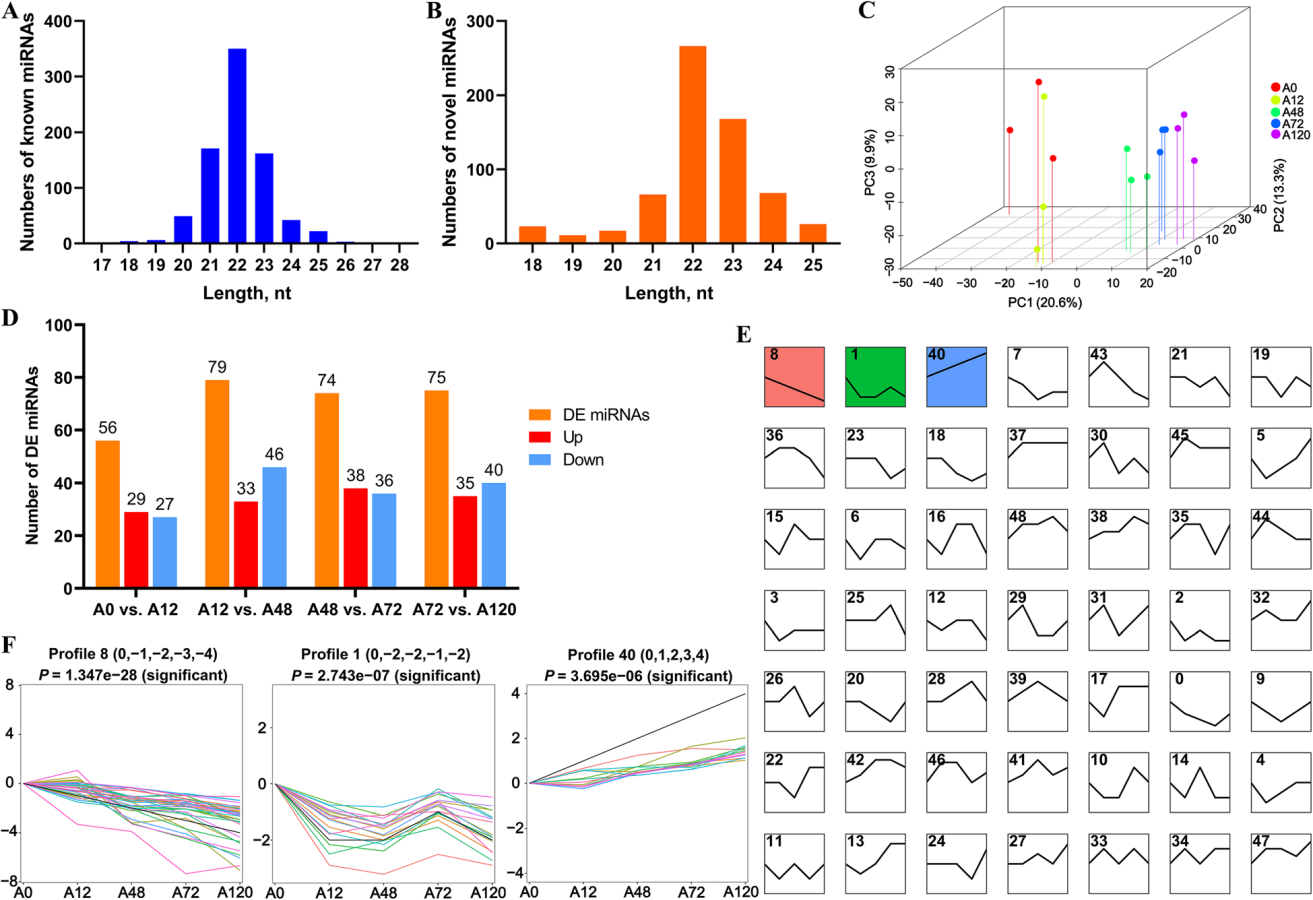
Characteristics and expression profiles of miRNAs in chicken abdominal adipocytes at various stages of differentiation. **A** Length distribution of known miRNA sequences in chicken abdominal adipocytes; **(B)** Length distribution of novel miRNA sequences in chicken abdominal adipocytes; **(C)** PCA analysis of the 15 samples, based on the normalized expression levels of all the expressed miRNAs; **(D)** Histogram analysis of the number of differentially expressed (DE)-miRNAs in four comparisons (A0 vs. A12, A12 vs. A48, A48 vs. A72, and A72 vs. A120); **(E)** Clustering of short time series expression of all DE-miRNAs; **(F)** Significant profiles (profiles 8, 1, and 40) identified via STEM analysis

In total, we obtained 190.21 million clean reads from 15 small RNA libraries, including the A0 (A0–1, A0–2, A0–3), A12 (A12–1, A12–2, A12–3), A48 (A48–1, A48–2, A48–3), A72 (A72–1, A72–2, A72–3), and A120 (A120–1, A120–2, A120–3) groups. The percentage of clean reads with a Phred quality score over 30 (Q30) ranged from 95.17% to 97.34% (Table 1). After filtering out the rRNA, tRNA, snRNA, snoRNA, and repeat-associated RNA sequences, the unannotated reads containing miRNAs were aligned to the Galgal 6.0 chicken reference genome to obtain mapped reads (mapping ratio: 70.87–79.43%). The 15 libraries contained 813 known miRNAs and 645 novel miRNAs. Both known miRNAs and novel miRNAs (20–24 nt in length) were abundant. The vast majority were 22 nt in length, which coincided with the typical range of length of miRNAs for Dicer-derived products (Fig. 1A, B). A principal component analysis also showed global differences among adipocytes at different differentiation stages (Fig. 1C). This suggested that our data were suitable for further analysis.

**Table 1:** Characteristics of the reads from 15 small RNA libraries for chicken adipocytes

Samples	Raw reads	Clean reads	GC, %	Q30, %	Unannotated reads	Mapped reads	Mapped ratio
A0–1	14,095,191	13,017,869	43.14	96.53	12,601,329	9,529,509	75.62%
A0–2	11,381,923	10,398,538	42.86	96.88	10,064,002	7,333,515	72.87%
A0–3	13,113,101	12,328,709	42.94	96.03	12,066,750	8,614,258	71.39%
A12–1	13,126,885	11,684,725	43.39	95.27	11,383,525	8,067,627	70.87%
A12–2	13,372,605	11,924,653	42.83	96.82	11,504,002	8,334,451	72.45%
A12–3	12,196,057	11,130,917	43.23	96.79	10,833,452	7,786,407	71.87%
A48–1	12,307,962	11,014,663	43.17	96.72	10,693,130	8,124,260	75.98%
A48–2	11,451,914	10,521,608	42.76	97.26	10,257,682	8,145,917	79.41%
A48–3	12,558,151	11,540,836	42.71	97.34	11,312,093	8,984,793	79.43%
A72–1	13,710,206	12,176,651	43.20	96.41	11,697,516	9,159,639	78.3%
A72–2	12,275,200	11,242,346	43.22	97.14	10,892,833	8,315,058	76.34%
A72–3	11,995,065	10,904,132	43.14	96.76	10,478,474	8,209,934	78.35%
A120–1	19,750,485	15,852,219	44.07	95.23	15,042,721	11,195,181	74.42%
A120–2	28,052,802	25,199,083	43.04	96.18	24,332,421	18,508,850	76.07%
A120–3	13,530,281	11,270,025	43.62	95.17	10,835,595	8,284,130	76.45%

### Differential expression profiles of miRNAs in chicken abdominal adipocytes

To identify the functional miRNAs that may play key regulatory roles in chicken adipogenesis, we screened DE-miRNAs during the adipogenic differentiation of chicken abdominal preadipocytes. A total of 217 DE-miRNAs were identified via four pairwise comparisons (including 56 in A0 vs. A12, 79 in A12 vs. A48, 74 in A48 vs. A72, and 75 in A72 vs. A120). Of these, 140 were known miRNAs and 77 were novel (Fig. 1D). These DE-miRNAs were clustered into 49 profiles, of which profiles 8, 1, and 40 were assigned a statistically significant number of genes (Fig. 1E, Additional File 1: Table S2). During adipogenic differentiation of chicken abdominal preadipocytes, profile 8—containing 35 DE-miRNAs—showed a gradually decreasing trend of expression. Profile 1—containing 18 DE-miRNAs—exhibited a trend of highest expression in preadipocytes, remarkably decreased expression at 12 and 48 h post-differentiation, moderately increased expression at 72 h post-differentiation, and decreased expression at 120 h post-differentiation. Profile 40—containing 14 DE-miRNAs—displayed a trend of sequentially upregulated expression during abdominal preadipocyte differentiation in chickens (Fig. 1F).

### Differential expression profiles of mRNAs in chicken abdominal adipocytes

**Fig. 2:**
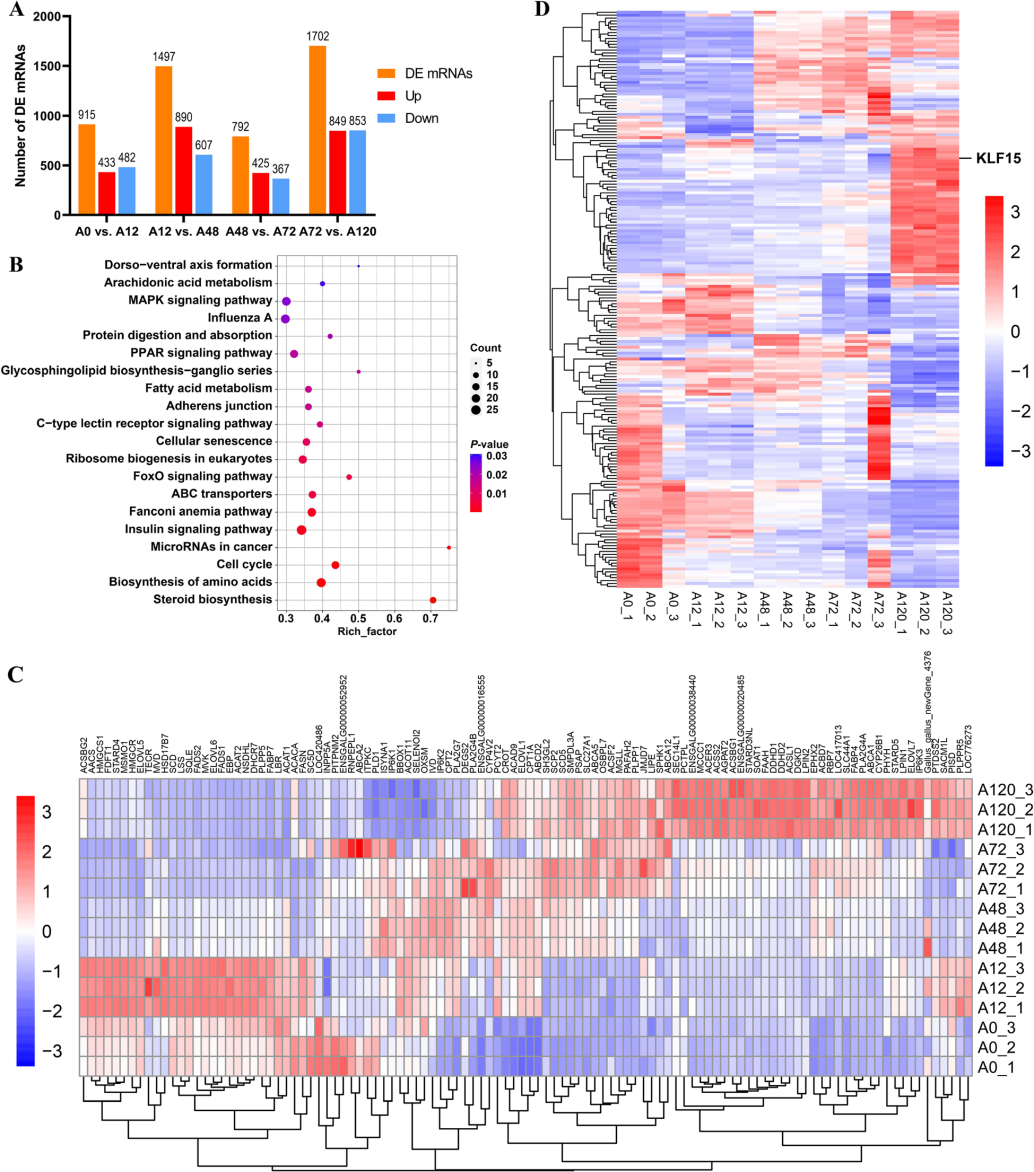
Expression profiles of mRNAs in chicken abdominal adipocytes at various stages of differentiation. **A** Histogram of the number of differentially expressed genes (DEGs) in four comparisons (A0 vs. A12, A12 vs. A48, A48 vs. A72, A72 vs. A120); **(B)** Top 20 pathways identified via Kyoto Encyclopedia of Genes and Genomes pathway enrichment analysis of all DEGs during adipogenic differentiation; **(C)** Heatmap of DEGs associated with lipid metabolism during adipogenesis; **(D)** Heatmap of differentially expressed transcriptional factors during adipogenesis

In total, we detected 3520 DEGs (including 915 in A0 vs. A12, 1497 in A12 vs. A48, 792 in A48 vs. A72, and 1702 in A72 vs. A120) comprising 3156 unigenes and 364 novel genes (Fig. 2A; Additional File 1: Table S3). Functional enrichment analysis showed that the DEGs were enriched in lipogenesis-related terms, such as cellular lipid metabolic process, regulation of cell differentiation, regulation of lipid metabolic process, positive regulation of lipid storage, positive regulation of lipid transport, regulation of lipid biosynthetic process, lipid metabolic process, lipid transport, and lipid homeostasis (Additional File 1: Table S4). Furthermore, several lipid-related signaling pathways, including the PPAR signaling pathway, MAPK signaling pathway, FoxO signaling pathway, steroid biosynthesis, and ABC transporters, were significantly enriched by DEGs (Fig. 2B; Additional File 1: Table S4). Of the DEGs associated with these enriched pathways, 110 were annotated to be strongly associated with lipid metabolism (Fig. 2C; Additional File 1: Table S3). The coding proteins of 198 DEGs were identified as transcriptional factors, wherein the *KLF15* gene was gradually upregulated with the progression of adipogenic differentiation in chicken abdominal preadipocytes (Fig. 2D; Additional File 1: Table S3).

### Integrative analysis of miRNA and mRNA expression profiles

**Fig. 3:**
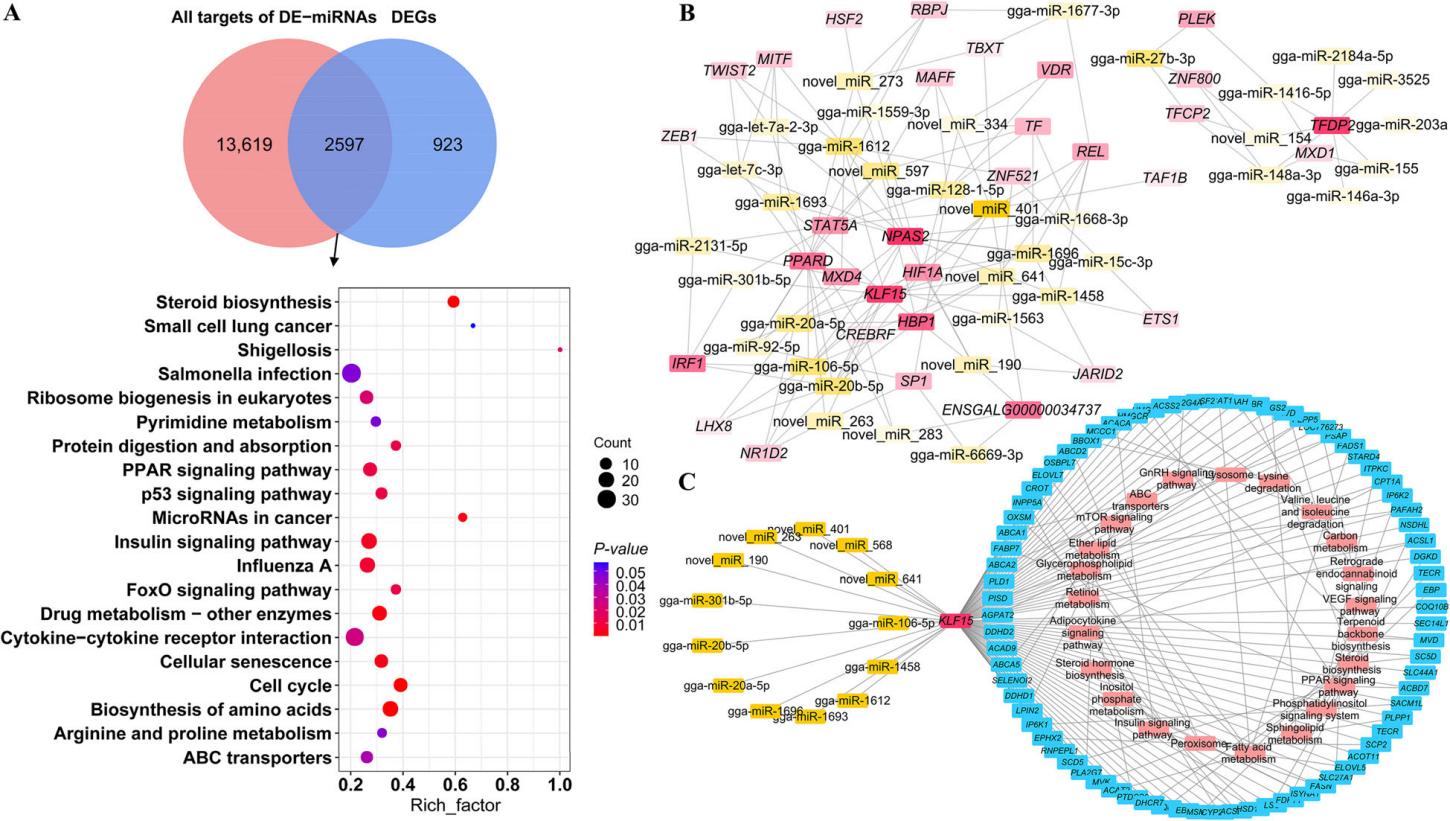
Integrative analysis of differentially expressed (DE)-miRNA and differentially expressed genes (DEGs). **A** Top 20 signaling pathways identified via Kyoto Encyclopedia of Genes and Genomes pathway enrichment analysis of differentially expressed targets of DE-miRNAs during adipogenic differentiation; **(B)** Regulatory networks involving DE-miRNAs and differentially expressed transcriptional factors; **(C)** Regulatory networks involving DE-miRNA, KLF15, DEGs related to lipid metabolism, and signaling pathways

To better understand the potential miRNAs responsible for adipogenesis in chickens, we conducted target prediction of DE-miRNAs and functional enrichment analysis. For 217 DE-miRNAs, a total of 16,216 target genes were identified. Of these, 2597 target genes were differentially expressed (Additional File 1: Table S5) and were significantly enriched in adipogenesis-related terms, including lipid transporter activity, lipid storage, lipid homeostasis, lipid transport, lipid droplets, and lipid biosynthetic processes (Additional File 1: Table S5). The significantly enriched lipid-related signaling pathways were the PPAR signaling pathway, FoxO signaling pathway, steroid biosynthesis, and ABC transporters (Fig. 3A; Additional File 1: Table S5).

In the present study, we focused on the miRNAs that potentially bind to the transcriptional factors involved in fat deposition. In total, we identified 2181 regulatory pairs involving 215 DE-miRNAs and 136 differentially expressed transcriptional factors. Of these, 271 regulatory pairs of DE-miRNAs and differentially expressed transcriptional factors were significantly negatively coexpressed (Additional File 1: Table S6). The regulatory network comprising the DE-miRNAs and differentially expressed transcriptional factors suggested that *KLF15* may be a crucial transcriptional factor that is regulated by 13 miRNAs during the adipogenic differentiation of chicken abdominal preadipocytes (Fig. 3B). A genome-wide search for KLF15 motifs indicated that 10,598 potential downstream genes, including 229 lipid-related genes, were potentially targeted by KLF15. The 229 lipid-related genes included 75 DEGs in the RNA-seq data (Additional File 1: Table S7). We constructed a regulatory network comprising the DE-miRNAs, *KLF15* gene, and lipid metabolism-related genes (Fig. 3C). We found that KLF15 may mediate the transcriptional regulation of representative genes closely related to lipid metabolism, such as sterol-C5-desaturase (*SC5D*), mevalonate diphosphate decarboxylase (*MVD*), ELOVL fatty acid elongase 5 (*ELOVL5*), fatty acid synthase (*FASN*), acyl-CoA synthetase bubblegum family member 2 (*ACSBG2*), acetyl-CoA carboxylase alpha (*ACACA*), acyl-CoA synthetase family member 2 (*ACSF2*), acyl-CoA synthetase long-chain family member 1 (*ACSL1*), fatty acid desaturase 1 (*FADS1*), stearoyl-CoA desaturase 5 (*SCD5*), 1-acylglycerol-3-phosphate O-acyltransferase 2 (*AGPAT2*), diglyceride acyltransferase 2 (*DGAT2*), apolipoprotein A4 (*APOA4*), and solute carrier family 27 member 1(*SLC27A1*, also known as *FATP*). By regulating these genes, KLF15 may affect several signaling pathways involved in lipid trafficking and, subsequently, adipogenesis. Additionally, the KLF15-mediated adipogenesis was blocked by post-transcriptional control of several miRNAs, including gga-miR-106-5p, which showed decreased expression in the mature adipocytes than in the preadipocytes (Additional File 1: Table S2).

### Dynamic expression patterns of adipocyte gga-miR-106-5p in vivo and in vitro

**Fig. 4:**
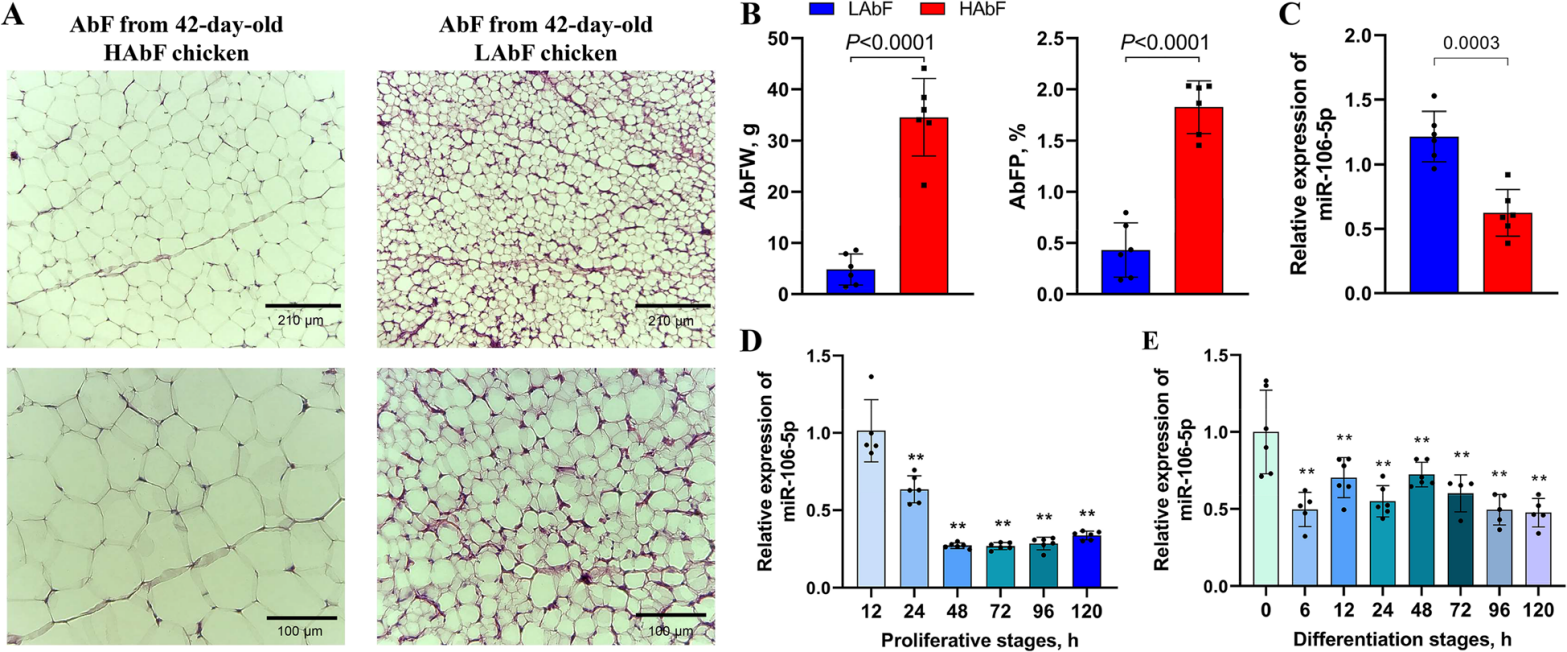
Expression patterns of adipocyte gga-miR-106-5p in vivo and in vitro. **A** Difference in volume between abdominal adipocytes in the abdominal fat tissues of chickens with high abdominal fat (HAbF) and low abdominal fat (LAbF), as determined via hematoxylin and eosin staining; **(B)** Difference in abdominal fat weight (AbFW) and abdominal fat percentage (AbFP) between HAbF and LAbF chickens; **(C)** Expression levels of gga-miR-106-5p in the abdominal fat tissues of HAbF and LAbF chickens; **(D)** Expression pattern of gga-miR-106-5p during abdominal preadipocyte proliferation in chickens; **(E)** Expression pattern of gga-miR-106-5p during adipogenic differentiation of chicken abdominal preadipocytes

To determine the expression profiles of gga-miR-106-5p in vivo and in vitro, we performed qRT-PCR analysis of abdominal fat in HAbF and LAbF chickens and of abdominal preadipocytes at different stages of proliferation and differentiation. The adipocyte diameter, abdominal fat weight, and abdominal fat percentage of HAbF broilers were distinctly higher than those of LAbF broilers (Fig. 4A, B). Compared with that in LAbF broilers, gga-miR-106-5p was significantly downregulated in the abdominal fat of HAbF broilers (Fig. 4C). Moreover, the expression levels of gga-miR-106-5p gradually decreased during abdominal preadipocyte proliferation in chickens (Fig. 4D) and were lower in mature adipocytes than in the preadipocytes (Fig. 4E). These results suggested that gga-miR-106-5p may play an important role in adipogenic differentiation in chickens.

### gga-miR-106-5p inhibits the proliferation of chicken abdominal preadipocytes

**Fig. 5:**
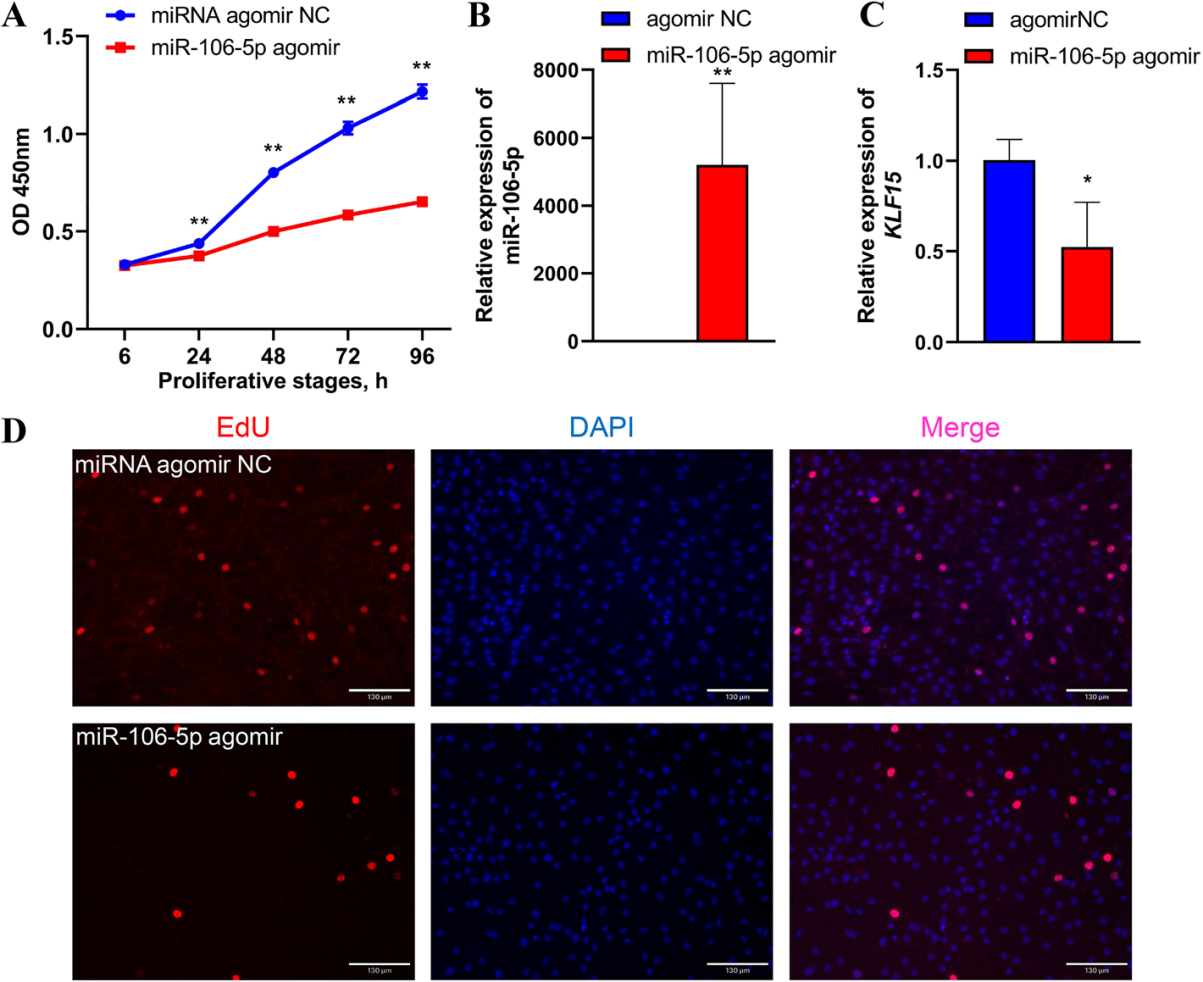
Effects of gga-miR-106-5p on abdominal preadipocyte proliferation in chickens. **A** CCK8 assay of chicken abdominal preadipocytes transfected with miR-106-5p agomir and the miR-106-5p agomir negative control (NC) at 12, 24, 48, 72, and 96 h post-transfection; **(B)** Detection of gga-miR-106-5p overexpression 48 h after transfecting miR-106-5p agomir in chicken abdominal preadipocytes; **(C)** Detection of the abundance of *KLF15* mRNA in chicken abdominal preadipocytes treated with miR-106-5p agomir and miR-106-5p agomir NC; **(D)** Representative images from the EdU assay of chicken abdominal preadipocytes transfected with miR-106-5p agomir and miR-106-5p agomir NC for 48 h

To confirm the effects of gga-miR-106-5p during cell proliferation, we transfected miR-106-5p agomir with ICP2 cells to overexpress gga-miR-106-5p. The CCK8 assay results demonstrated that the overexpression of gga-miR-106-5p at 24, 48, 72, and 96 h significantly reduced the number of living cells compared with that in the NC (Fig. 5A). The marked overexpression of gga-miR-106-5p (by approximately 6600-fold) in miR-106-5p agomir-treated ICP2 cells was confirmed via qRT-PCR (Fig. 5B), and it resulted in the reduced mRNA expression of *KLF15* (Fig. 5C). Moreover, the EdU assay revealed significantly fewer EdU-positive cells in the miR-106-5p agomir group than in the NC group (Fig. 5D). This indicated that gga-miR-106-5p inhibited abdominal preadipocyte proliferation in chickens.

### gga-miR-106-5p inhibits the differentiation of chicken abdominal preadipocytes

**Fig. 6:**
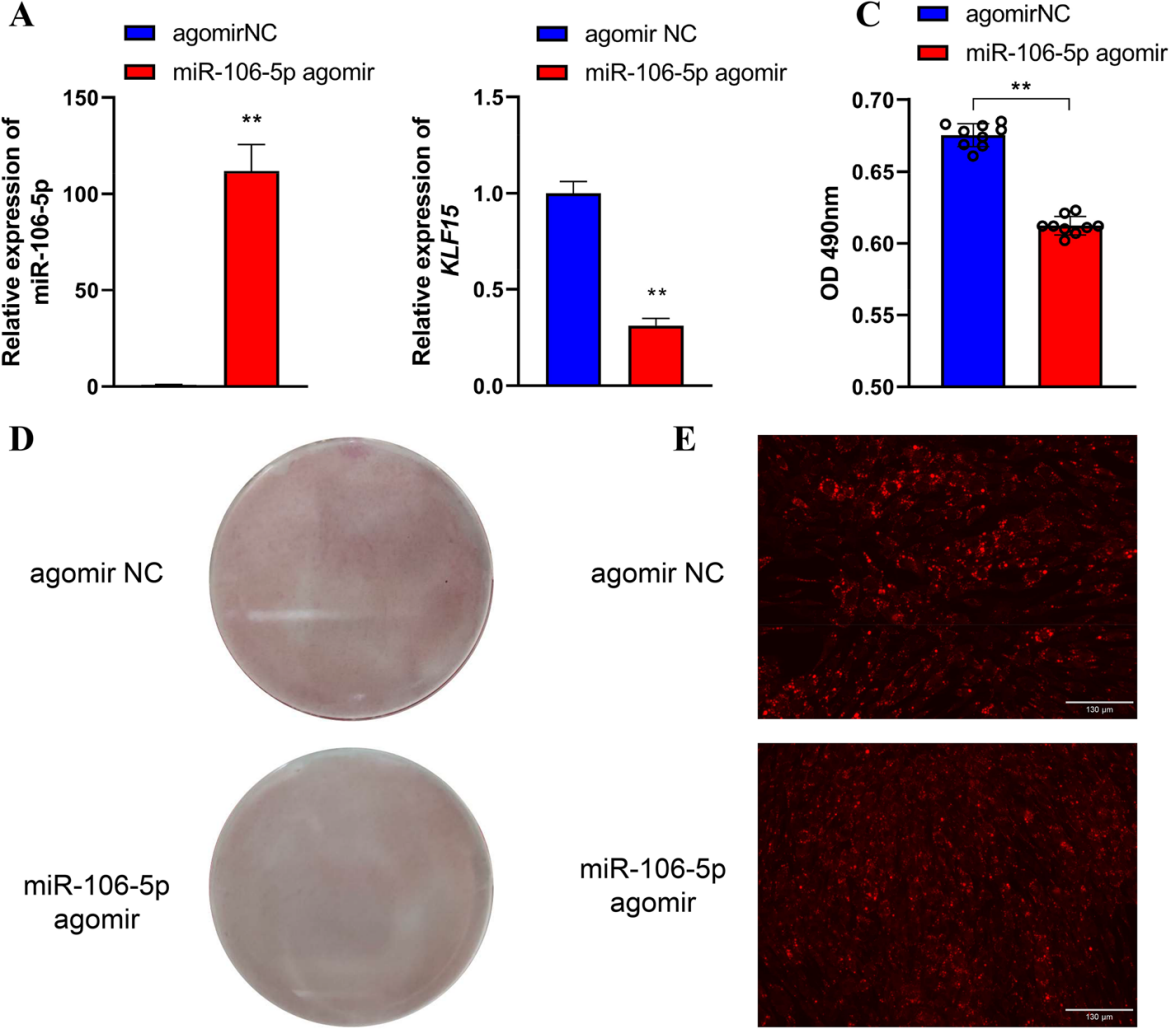
Effects of gga-miR-106-5p on the adipogenic differentiation of chicken abdominal preadipocytes. **A** Detection of miR-106-5p overexpression after transfecting miR-106-5p agomir into the differentiated abdominal adipocytes of chicken; **(B)** Detection of the abundance of *KLF15* mRNA in the differentiated abdominal adipocytes of chicken treated with miR-106-5p agomir and miR-106-5p agomir negative control (NC); **(C)** Spectrophotometric analysis of lipid droplet content via Oil red O staining of the differentiated chicken abdominal adipocytes transfected with miR-106-5p agomir and miR-106-5p agomir NC; **(D)** Oil red O staining of the differentiated chicken abdominal adipocytes transfected with miR-106-5p agomir and miR-106-5p agomir NC; **(E)** Representative images of Nile red fluorescent staining of the differentiated chicken abdominal adipocytes transfected with miR-106-5p agomir and miR-106-5p agomir NC

To elucidate the functions of gga-miR-106-5p in preadipocyte differentiation in chickens, we treated ICP2 cells with miR-106-5p agomir to overexpress gga-miR-106-5p. Following this, we induced adipogenesis in cells through exposure to 160 μmol/L sodium oleate. After adipogenic differentiation for 48 h, the accumulation of lipid droplets was visualized by staining with Oil Red O and the lipophilic fluorophore Nile red. qRT-PCR analysis revealed that compared with the NC, miR-106-5p agomir-treated ICP2 cells showed remarkably high expression of gga-miR-106-5p (by approximately 112-fold). This resulted in a significant decrease in the expression of *KLF15* mRNA (Fig. 6A, B). The significantly reduced lipid droplet content of miR-106-5p agomir-treated ICP2 cells was validated by staining with Oil Red O (Fig. 6C, D) and Nile red (Fig. 6E).

### The *KLF15* gene is a direct target of gga-miR-106-5p

**Fig. 7:**
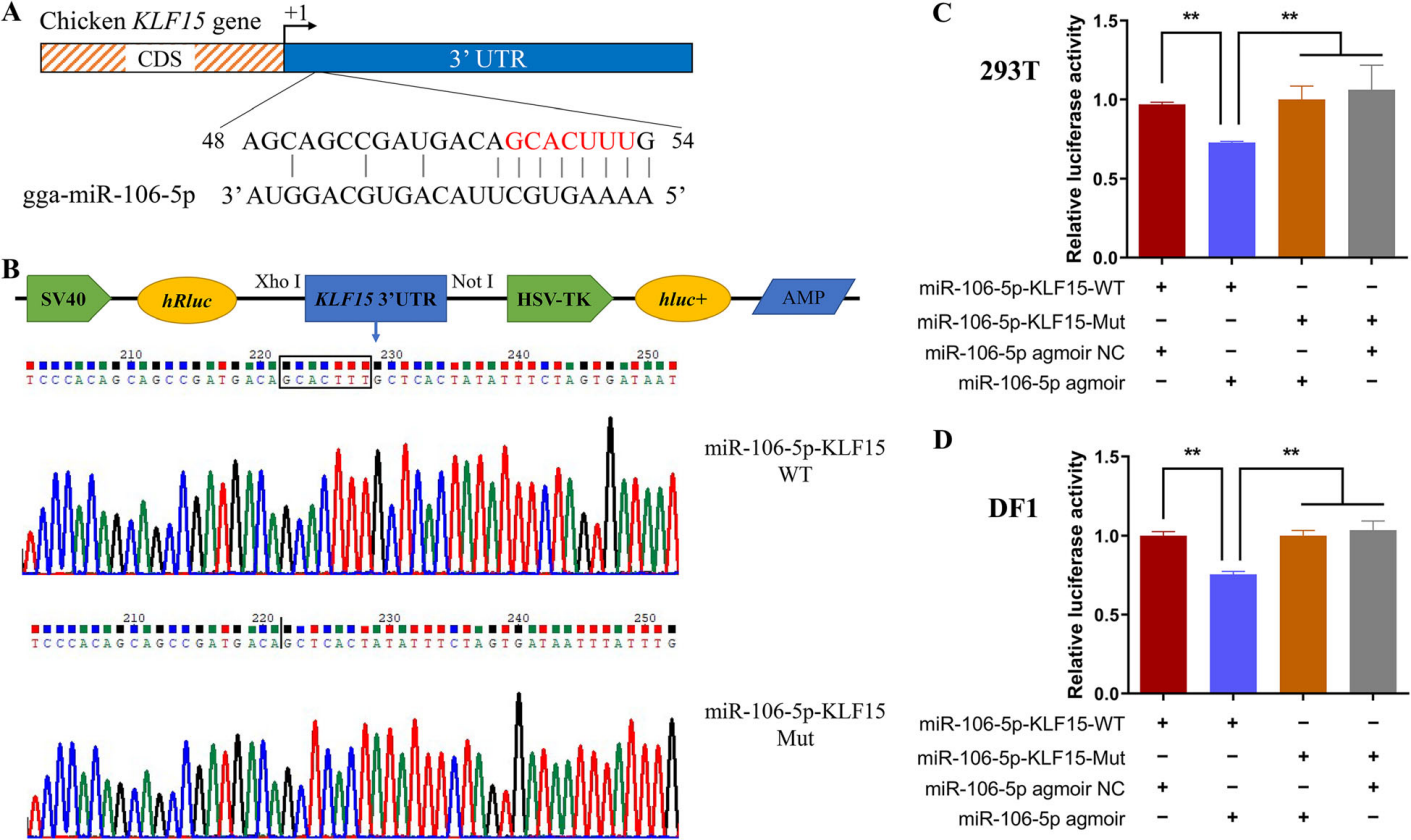
Validation of the *KLF15* gene as a direct target of gga-miR-106-5p. **A** Potential gga-miR-106-5p binding sites in the 3′ UTR of the *KLF15* gene; **(B)** Construction and validation of dual-luciferase reporter vectors for the validation of gga-miR-106-5p targeting the *KLF15* gene. The sequences with or without the binding sites of gga-miR-106-5p and the 3′ UTR of the *KLF15* gene were cloned into the psiCHECK-2 vector. WT: wild-type vector; mut: mutant vector; *hRluc*: *Renilla* luciferase; *hluc+*: firefly luciferase; **(C, D)** Validation of the interaction between gga-miR-106-5p and the 3′ UTR of the *KLF15* gene via a dual-luciferase reporter assay in 293 T cells and DF1 cells. Data are presented as mean ± SD (*n* = 3)

Our results suggested that gga-miR-106-5p could bind to the 3′ UTR region of the *KLF15* gene (Fig. 7A). To confirm the interaction between gga-miR-106-5p and *KLF15*, we constructed wild-type and mutant plasmids that contained and lacked the gga-miR-106-5p binding sites in the 3′ UTR of the *KLF15* gene, respectively. The plasmids were successfully co-transfected with the miR-106-5p agomir or NC into DF1 cells and 293 T cells (Fig. 7B). As expected, a dual-luciferase reporter assay revealed that gga-miR-106-5p significantly inhibited the relative luciferase activity of the wild-type KLF15 reporter vector (miR-106-5p-KLF15-WT), but did not affect the relative luciferase activity of the mutant KLF15 reporter vector (miR-106-5p-KLF15-Mut) in either DF1 or 293 T cells (Fig. 7C). These results indicate that the *KLF15* gene is a direct target of gga-miR-106-5p.

### Dynamic expression patterns of the adipocyte *KLF15* gene in vivo and in vitro

**Fig. 8:**
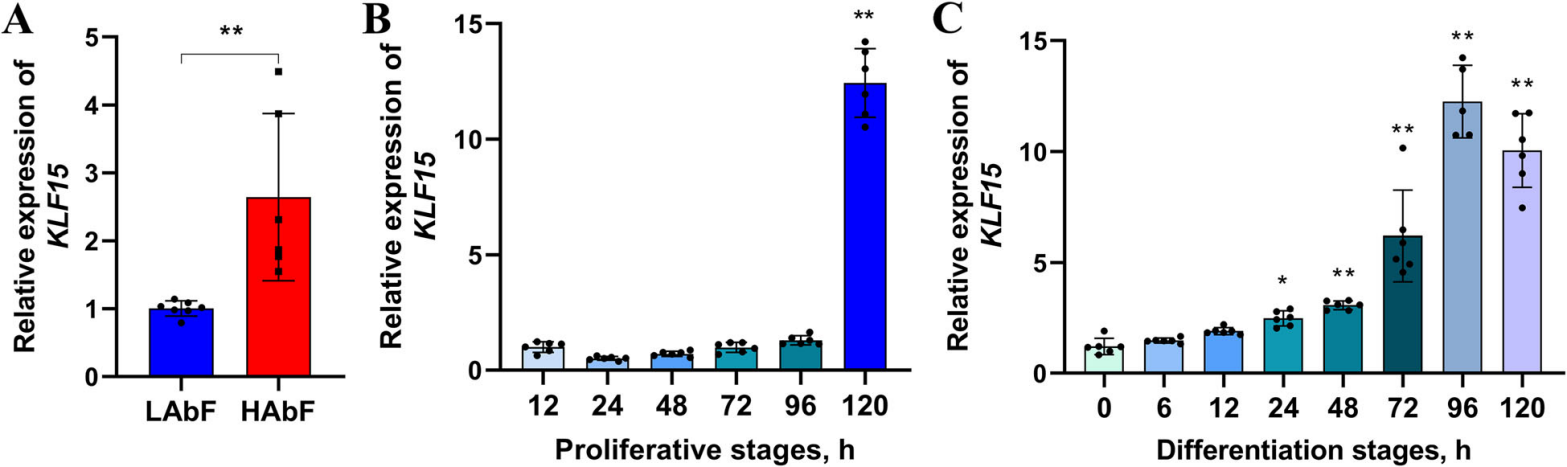
Expression patterns of the *KLF15* gene in adipocytes in vivo and in vitro. **A** mRNA expression levels of *KLF15* in the abdominal fat tissues of chickens with high (HAbF) and low (LAbF) abdominal fat; **(B)** mRNA expression pattern of *KLF15* during the proliferation of chicken abdominal preadipocytes; **(C)** mRNA expression pattern of *KLF15* during the adipogenic differentiation of chicken abdominal preadipocytes

To determine whether *KLF15* is involved in abdominal adipogenic differentiation in chickens, we evaluated the differences in *KLF15* mRNA expression between the abdominal fat of HAbF and LAbF broilers and in chicken abdominal preadipocytes at different stages of proliferation and differentiation. We found that the mRNA expression of *KLF15* was remarkably higher in the abdominal fat of HAbF broilers than in that of LAbF broilers (Fig. 8A). In addition, the abundance of *KLF15* mRNA increased gradually at different stages of proliferation and differentiation (Fig. 8B, C).

### KLF15 promotes the proliferation of chicken abdominal preadipocytes

**Fig. 9:**
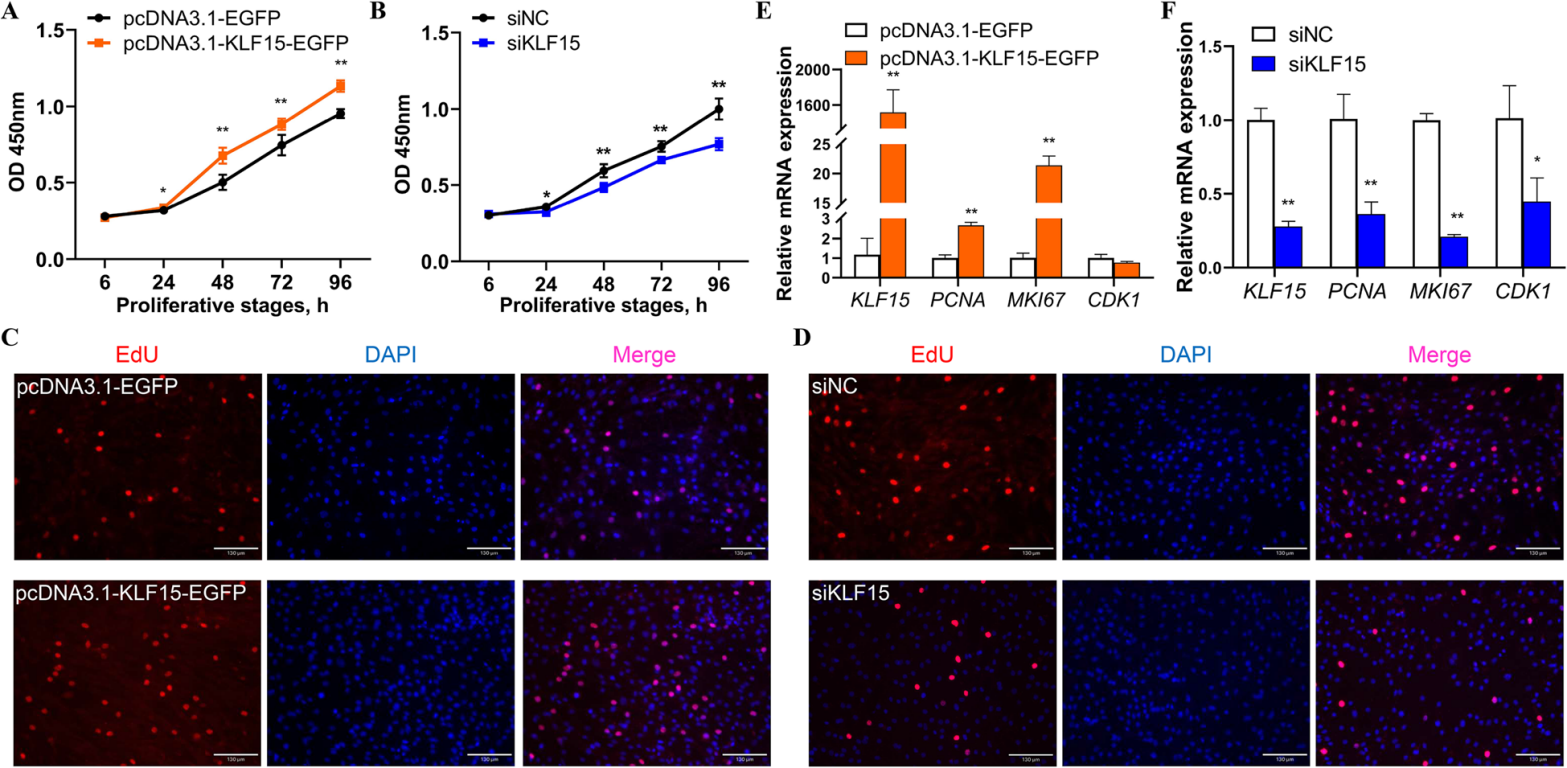
Effects of *KLF15* on the proliferation of chicken abdominal preadipocytes. **A** CCK8 assay of chicken abdominal preadipocytes transfected with pcDNA3.1-KLF15-EGFP and pcDNA3.1-EGFP at 12, 24, 48, 72, and 96 h post-transfection; **(B)** CCK8 assay of chicken abdominal preadipocytes transfected with siKLF15 and siNC at 12, 24, 48, 72, and 96 h post-transfection; **(C)** Representative images from the EdU assay of chicken abdominal preadipocytes transfected with pcDNA3.1-EGFP and pcDNA3.1-KLF15-EGFP at 48 h post-transfection; **(D)** Representative images from the EdU assay of chicken abdominal preadipocytes transfected with siNC and siKLF15 at 48 h post-transfection; **(E)** mRNA expression levels of *KLF15*, *PCNA*, *MKI67*, and *CDK1* after *KLF15* overexpression; **(F)** mRNA expression levels of *KLF15*, *PCNA*, *MKI67*, and *CDK1* after *KLF15* knockdown

To verify the regulatory role of KLF15 in chicken adipogenesis, we monitored the abdominal preadipocyte proliferation in ICP2 cells with *KLF15* overexpression and *KLF15* knockdown in chickens. The CCK8 assay demonstrated that compared with the pcDNA3.1-EGFP-treated control group, the KLF15 overexpression group had a significantly high number of living cells (Fig. 9A). In contrast, compared with the siNC control group, the siKLF15 knockdown group had a significantly low number of living cells (Fig. 9B). Furthermore, the EdU assay demonstrated a higher number of EdU-positive cells in the KLF15 overexpression group and a lower number of EdU-positive cells in the KLF15 knockdown group (Fig. 9C, D). Compared with that in the pcDNA3.1-EGFP-treated control group, the mRNA expression level of *KLF15* was significantly increased in the KLF15 overexpression group (Fig. 9E). The marker genes related to cell proliferation—including proliferating cell nuclear antigen (*PCNA*), marker of proliferation Ki-67 (*MKI67*), and cyclin dependent kinase 1 (*CDK1*)—possessed putative KLF15 binding sites in their promoters (Additional File 1: Table S8). The mRNA expression levels of *PCNA* and *MKI67* were significantly higher in the *KLF15* overexpression group than in the pcDNA3.1-EGFP-treated control group (Fig. 9E). In contrast, the mRNA expression levels of *KLF15* and the marker genes involved in cell proliferation—including *PCNA*, *MKI67*, and *CDK1*—were significantly lower in the siKLF15 knockdown group than in the siNC control group (Fig. 9F). These results suggest that KLF15 positively regulates abdominal preadipocyte proliferation in chickens.

### KLF15 facilitates the differentiation of chicken abdominal preadipocytes

**Fig. 10:**
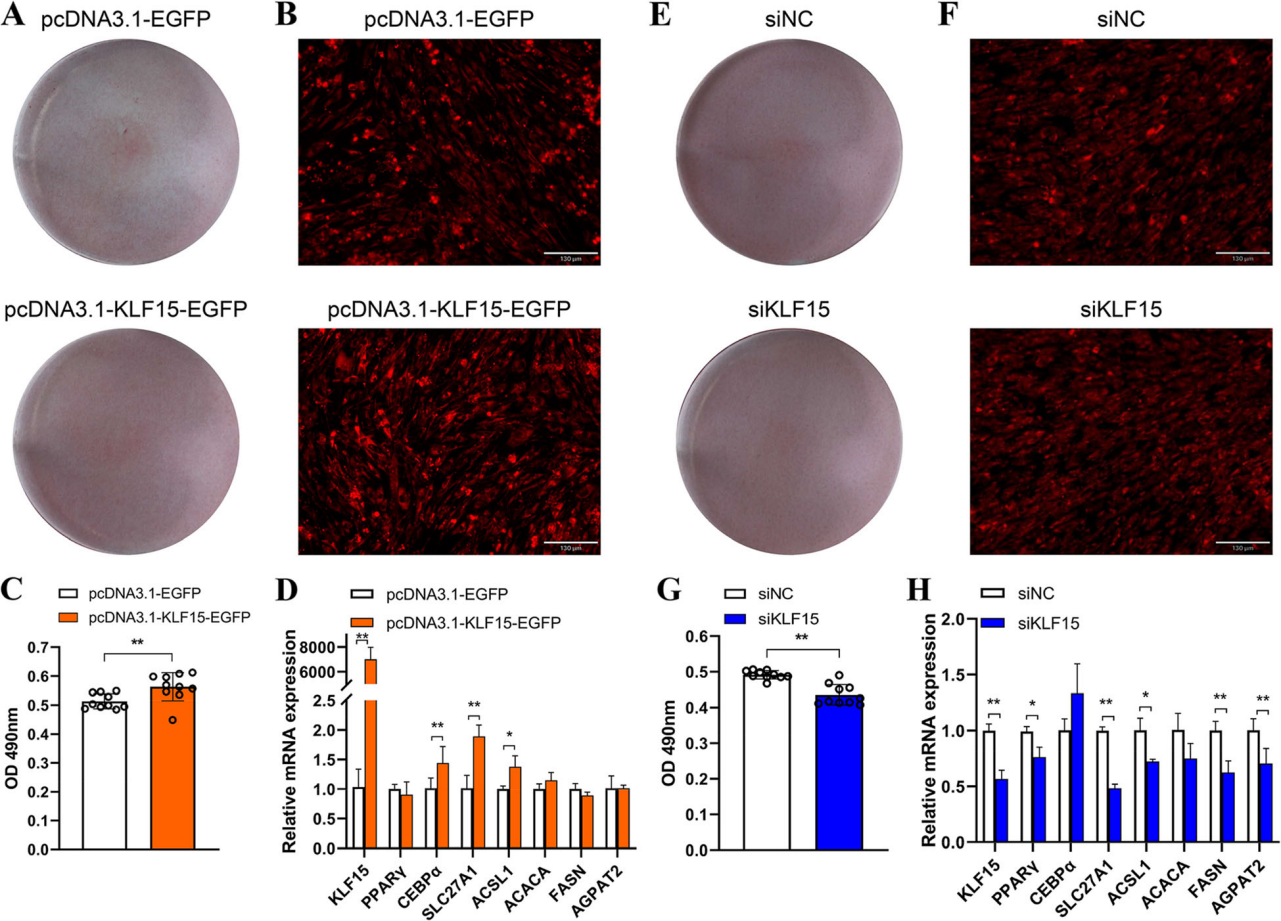
Effects of *KLF15* on the adipogenic differentiation of chicken abdominal preadipocytes. **A** Oil red O staining of the differentiated chicken abdominal adipocytes transfected with pcDNA3.1-EGFP and pcDNA3.1-KLF15-EGFP; **(B)** Representative images of Nile red fluorescent staining of the differentiated chicken abdominal adipocytes transfected with pcDNA3.1-EGFP and pcDNA3.1-KLF15-EGFP; **(C)** Spectrophotometric analysis of lipid droplet content via Oil red O staining of the differentiated chicken abdominal adipocytes transfected with pcDNA3.1-EGFP and pcDNA3.1-KLF15-EGFP; **(D)** mRNA expression levels of *KLF15*, *PPARγ*, *CEBPα*, *SLC27A1*, *ACSL1*, *ACACA*, *FASN*, and *AGPAT2* in the differentiated chicken abdominal adipocytes transfected with pcDNA3.1-EGFP and pcDNA3.1-KLF15-EGFP; **(E)** Oil red O staining of the differentiated chicken abdominal adipocytes transfected with siNC and siKLF15; **(F)** Representative images of Nile red fluorescent staining of the differentiated chicken abdominal adipocytes transfected with siNC and siKLF15; **(G)** Spectrophotometric analysis of lipid droplet content via Oil red O staining of the differentiated chicken abdominal adipocytes transfected with siNC and siKLF15; **(H)** mRNA expression levels of *KLF15*, *PPARγ*, *CEBPα*, *SLC27A1*, *ACSL1*, *ACACA*, *FASN*, and *AGPAT2* in the differentiated chicken abdominal adipocytes transfected with siNC and siKLF15

To further assess the regulatory role of KLF15 in chicken adipogenesis, we monitored the changes in lipid droplet accumulation in differentiated ICP2 cells with *KLF15* overexpression and *KLF15* knockdown. After adipogenic differentiation for 48 h, excessive *KLF15* expression induced a significant increase in lipid droplet content, as determined with the Oil Red O and Nile red fluorescent staining assays (Fig. 10A–C). The lipogenesis-related genes, including *PPARγ*, CCAAT/enhancer-binding protein *α* (*CEBPα*), *SLC27A1*, *ACSL1*, *ACACA*, *FASN*, and *AGPAT2*, were equipped with putative KLF15 binding sites in their promoters (Additional File 1: Table S8). Accordingly, the *KLF15*, *CEBPα*, *SLC27A1*, and *ACSL1* genes were significantly induced upon *KLF15* stimulation (Fig. 10D). In contrast, lipid droplet content was significantly lower in the siKLF15 knockdown group than in the siNC control group (Fig. 10E–G). The mRNA expression of *KLF15*, *PPARγ*, *SLC27A1*, *ACSL1*, *FASN*, and *AGPAT2* was remarkably decreased in the siKLF15 knockdown group compared to that in the siNC control group (Fig. 10H). These results indicate that KLF15 may accelerate abdominal preadipocyte differentiation in chickens.

## Discussion

In recent years, investigations into the regulatory mechanisms underlying abdominal fat deposition in mammals have increased considerably. miRNAs are key regulators of gene expression at the post-transcriptional level and are known to play important roles in a wide range of biological processes. Thus, their emerging regulatory role in adipogenesis has attracted much interest. In the present study, we constructed dynamic expression profiles of miRNAs and mRNAs in chicken abdominal adipocytes at different stages of differentiation. We identified a total of 1458 miRNAs (comprising 813 known miRNAs and 645 novel miRNAs), which expands the number of miRNAs known to be expressed in the abdominal adipocytes of chickens. Several miRNAs—including gga-miR-19b-3p, gga-miR-206, and gga-miR-20a-5p—that functionally affect preadipocyte proliferation or differentiation in chickens were also differentially expressed in our study [18, 19, 21]. The differentially expressed mRNAs were significantly enriched in several signaling pathways associated with lipid metabolism, including the PPAR signaling pathway, MAPK signaling pathway, FoxO signaling pathway, steroid biosynthesis, and ABC transporters. Interestingly, the PPAR signaling pathway is an emblematic intermediate metabolic pathway that mediates the regulation of adipogenesis, wherein PPARs (in conjunction with their activating ligands) are necessary and sufficient to initiate an adipogenic program [8, 37]. Simultaneously, the differentially expressed targets of differentially expressed miRNAs were significantly involved in these signaling pathways, including the PPAR signaling pathway, FoxO signaling pathway, steroid biosynthesis, and ABC transporters. These results suggested that miRNAs were indeed responsible for the adipogenic differentiation of abdominal preadipocytes in chickens.

Extensive research has suggested that the miR-106 family may serve as a crucial regulator of intracellular physiological processes including cell proliferation, apoptosis, and migration in cancer [38–41]. Moreover, miR-106a is known to promote the adipogenic differentiation of adipose-derived mesenchymal stem cells [42]. Therefore, we speculated that miR-106-5p—a member of the miR-106 family—is responsible for the proliferation and differentiation of abdominal preadipocytes in chickens. Our analysis of the expression pattern of gga-miR-106-5p indicated that its expression was downregulated in the abdominal fat tissues of chickens with a high abdominal fat percentage. In addition, gga-miR-106-5p was associated with an overall decrease in the proliferation and adipogenic differentiation of abdominal preadipocytes in chickens. The overexpression of gga-miR-106-5p in abdominal preadipocytes contributed to a conspicuous decrease in cell proliferation, which was accompanied by a significant decrease in lipid droplet content during abdominal preadipocyte differentiation. This indicates that gga-miR-106-5p may serve as an inhibitor of adipogenesis, resulting in the proliferation and differentiation of abdominal preadipocytes in chickens.

miRNAs are known to exert their effects via post-transcriptional silencing of the downstream target genes. In the present study, the *KLF15* gene was validated as a direct target of gga-miR-106-5p, whose 2–8 nt seed region at the 5′ end could recognize and bind to *KLF15* (as determined by a dual-luciferase reporter assay). KLF15 is a member of the KLF subclass of zinc finger transcription factors that bind to CACCC- and GC-rich DNA sequences within promoters. KLF15 has been implicated in lipid metabolism, thereby gaining prominence as a key adipogenic driver [43]. KLF15 is present in the nuclei of white adipocytes in mice, and its overexpression in 3T3-L1 cells potently induces adipocyte maturation [44]. Moreover, the functional evaluation of KLF15 in adipocytes differentiated in vitro has revealed that KLF15 knockdown inhibits insulin-stimulated lipogenesis in the adipocytes isolated from subcutaneous white adipose tissue [45]. Adipose-specific KLF15 knockout mice showed decreased adiposity, including lower white adipose tissue weight and fat composition, along with a nearly 30% decrease in adipocyte diameter in both epididymal and inguinal white adipose tissues. Similarly, the suppression of KLF15 in the differentiated 3T3-L1 cells significantly reduced their lipid content and enhanced lipolysis, and vice versa [46, 47]. These findings indicate that KLF15 is a potent regulator of adipogenesis in mammals. However, its role in chickens has remained largely unknown, and few studies have investigated its functions in lipid metabolism.

Initial insights linking the *KLF15* gene to economic traits were gleaned from a study reporting that KLF15 might function during the early stages of embryonic development owing to its restricted expression patterns in chickens [48]. Single nucleotide polymorphisms of the *KLF15* gene were found to be significantly associated with chicken growth and carcass traits [49]. A study also reported that KLF15 may participate in intramuscular fat deposition in Tibetan chickens [50]. Moreover, the mRNA and protein expression levels of KLF15 were reported to increase during preadipocyte differentiation in chickens [51]. Compared to that in preadipocytes, the *KLF15* gene was found to be significantly upregulated in mature adipocytes derived from intramuscular and abdominal fat tissues [52]. These findings led researchers to speculate that the *KLF15* gene plays an indispensable role in adipogenesis in chickens. Our findings revealed that the *KLF15* mRNA level was significantly higher in the abdominal fat of chickens with high abdominal fat percentage than that in chickens with low abdominal fat percentage, which is the opposite of the expression pattern of gga-miR-106-5p. Moreover, *KLF15* gene expression was significantly upregulated during abdominal preadipocyte differentiation in chickens, agreeing with the results of previous studies on the mature abdominal adipocytes of chickens [52] and differentiated 3T3-L1 cells of mammals [44, 47]. Thus, we speculated that KLF15 could positively regulate the adipogenic differentiation of abdominal preadipocytes in chickens. Our in-depth experiments examining the gain- and loss-of-function of the *KLF15* gene demonstrated that the *KLF15* gene could also facilitate adipogenic differentiation, as evidenced by increased lipid droplet accumulation in the abdominal preadipocytes of chickens. This was consistent with the results of the above-mentioned research on mammals showing that KLF15 functions as an activator in adipogenesis.

It has been suggested that *KLF15* mediates lipid metabolism and functions as an activator of adipogenesis by regulating the dynamic expression of key lipid-related genes in mammals [47]. To further investigate the regulatory mechanism through which KLF15 contributes to the adipogenic differentiation of abdominal preadipocytes in chickens, we predicted the downstream genes of KLF15 that contained putative KLF15 binding sites within their promoters (since KLF15 is a transcriptional factor). Several lipid-related genes have been identified as potential targets of KLF15. For example, *PPARγ* belongs to the nuclear receptor superfamily of ligand-activated transcription factors and is necessary and sufficient for adipocyte differentiation [53]. *PPARγ* transcriptional levels in abdominal fat tissue are significantly positively correlated with abdominal fat deposition in chickens. Moreover, knockdown of the *PPARγ* gene in the abdominal preadipocytes of chickens suppresses cell differentiation, as evidenced by reduced lipid droplet accumulation [54, 55]. Our result demonstrated that a significant decrease of *PPARγ* expression was triggered by KLF15 knockdown in chicken abdominal adipocytes, which was consistent with the previous study in mammals [47]. SLC27A1 is an integral membrane protein that accelerates long-chain fatty acid influx. A decrease in SLC27A1 expression can facilitate intramuscular fat deposition and prevent abdominal preadipocyte differentiation in chickens [56, 57]. Interestingly, the bovine *SLC27A1* gene is highly expressed in subcutaneous adipose tissue, and its transcriptional activity is dependent on the KLF15 transcription factor, which binds to the promoter region to drive *SLC27A1* transcription [58]. Agreeing with this, we also found that KLF15 could induce the transcriptional expression of the *SLC27A1* gene in chicken abdominal adipocytes. FASN is a critical metabolic enzyme for lipogenesis that catalyzes the synthesis of intracellular saturated fatty acids. The inhibition of FASN can prevent the differentiation of 3T3-L1 cells and repress lipid accumulation, which suggests that FASN is an active and essential component to maintain preadipocyte differentiation [59, 60]. Our result showed that KLF15 downregulation responded to the inhibited *FASN* expression, cohering with the previous findings about hepatic KLF15 in mice [61]. Additionally, the transcriptional activities of *CEBPα* (a crucial determinant of adipocyte fate) and the *ACSL1* and *AGPAT2* genes (responsible for de novo lipogenesis) were positively regulated by KLF15 in the abdominal adipocytes of chickens. Beyond that, some genes associated with cell proliferation—including *CDK1*, *PCNA*, and *MKI67*—were positively regulated by *KLF15*. Therefore, we hypothesized that during adipogenesis in chickens, KLF15 may serve as an activator in a manner that affects the expression of genes related to lipogenesis and cell proliferation, thus influencing the hyperplasia of abdominal preadipocytes and their differentiation into mature adipocytes. These regulatory mechanisms of KLF15 underlying adipogenesis need to be further verified.

Taken together, our miRNA-seq results indicate that a candidate miRNA, gga-miR-106-5p, is involved in the adipogenic differentiation of abdominal preadipocytes in chickens. Bioinformatic analysis and functional assays revealed that gga-miR-106-5p inhibits adipogenesis in chicken abdominal preadipocytes by targeting the *KLF15* gene. However, given that the *KLF15* gene was potentially regulated by more known and novel miRNAs, our results imply that gga-miR-106-5p may not be the sole mediator of the *KLF15* gene to inhibit chicken abdominal preadipocyte proliferation and differentiation, and its inhibitory role in abdominal adipogenesis should probably be accounted for by multiple targets in chickens.

## Conclusions

**Fig. 11:**
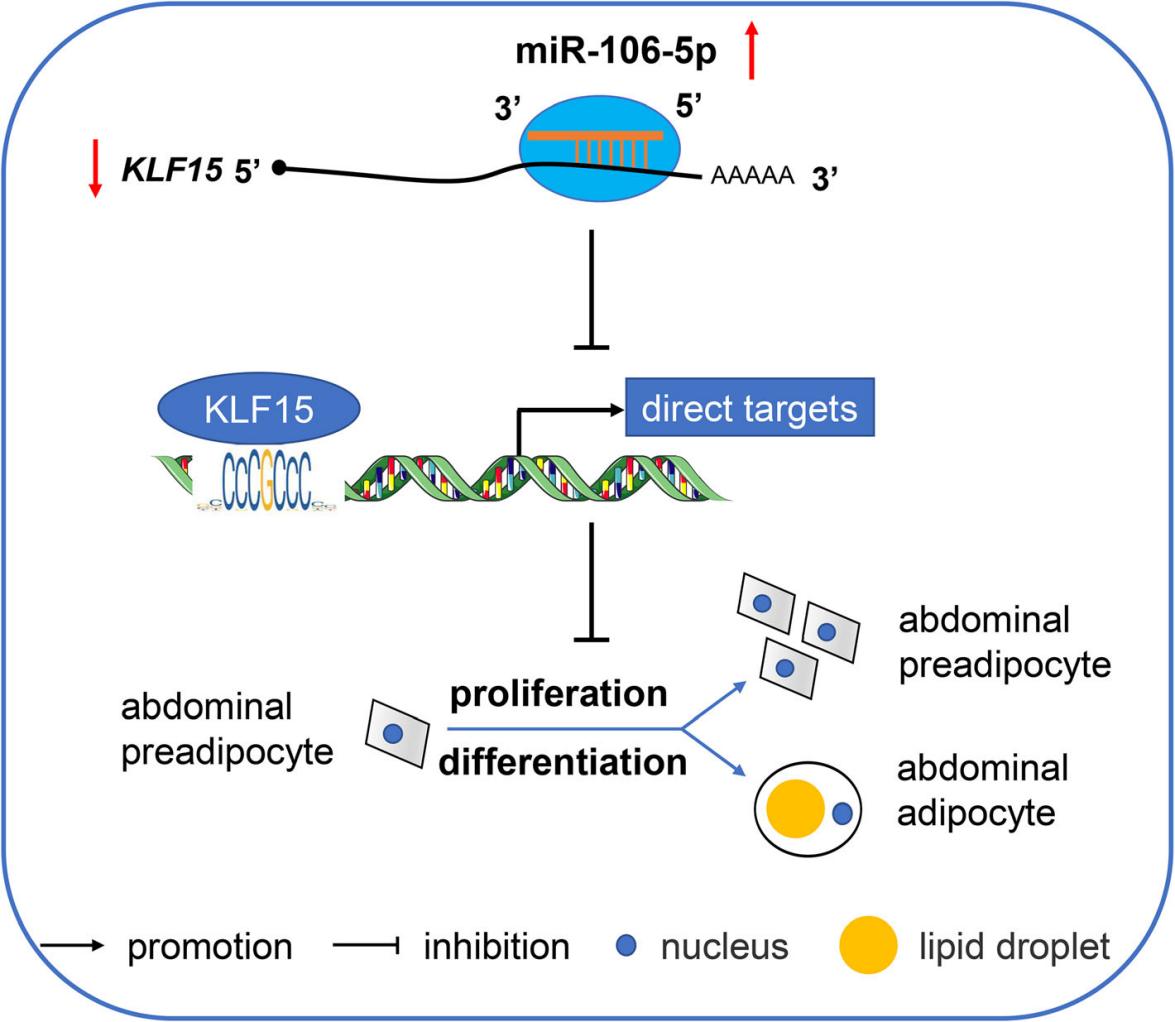
Proposed model of gga-miR-106-5p regulation of abdominal adipogenesis in chicken

We identified the miRNAs involved in chicken adipogenesis and showed that a candidate miRNA, gga-miR-106-5p, was closely associated with abdominal preadipocyte proliferation and adipogenic differentiation in chickens. Functional and regulatory analyses showed that gga-miR-106-5p could inhibit the proliferation and differentiation of chicken abdominal preadipocytes by targeting the *KLF15* gene. Overall, gga-miR-106-5p serves as a negative regulator of chicken adipogenesis by targeting *KLF15* (Fig. 11). Coupled with previous findings on the regulatory roles of miRNAs and the *KLF15* gene in adipogenesis, we propose that gga-miR-106-5p and *KLF15* may be crucial targets for the genetic improvement of excessive abdominal fat deposition in chickens.

## Supplementary Information

**Additional file 1: Table S1.** Primers used in this study. **Table S2.** Clustering of differentially expressed miRNA expression profiles in chicken abdominal adipocytes at different stages of differentiation. **Table S3.** Differentially expressed genes during the adipogenic differentiation of chicken abdominal preadipocytes. **Table S4.** Functional enrichment analysis of all differentially expressed mRNAs in chicken abdominal adipocytes at different stages of differentiation. **Table S5.** All targets of differentially expressed miRNAs and functional enrichment analysis of differentially expressed targets. **Table S6.** Regulatory network analysis of differentially expressed miRNAs and differentially expressed transcriptional factors. **Table S7.** Potential target genes of the KLF15 transcriptional factor. **Table S8.** Putative chicken KLF15 motif in the promoters of representative genes related to cell proliferation and lipogenesis.

## Data Availability

The data for the current study are available from the corresponding author upon reasonable request.

## Abbreviations

AbFW: Abdominal fat weight
AbFP: Abdominal fat percentage
HAbF: High abdominal fat percentage
LAbF: Low abdominal fat percentage
RNA-seq: High-throughput sequencing of RNA
qRT-PCR: Quantitative real-time PCR
miRNAs: MicroRNAs
DE-miRNAs: Differentially expressed miRNAs
DEGs: Differentially expressed genes
3′ UTRs: 3′ untranslated regions
TSS: Transcription start site
ICP2: Immortalized chicken preadipocytes 2
DMEM-F12: Dulbecco’s modified Eagle’s medium F12
FBS: Fetal bovine serum
cDNA: Complementary DNA
CCK8: Cell Counting Kit-8
EdU: 5-Ethynyl-2-deoxyuridine
KLF15: Kruppel like factor 15
PPARγ: Peroxisome proliferator activated receptor gamma
SC5D: Sterol-C5-desaturase
MVD: Mevalonate diphosphate decarboxylase
ELOVL5: ELOVL fatty acid elongase 5
FASN: Fatty acid synthase
ACSBG2: Acyl-CoA synthetase bubblegum family member 2
ACACA: Acetyl-CoA carboxylase alpha
ACSF2: Acyl-CoA synthetase family member 2
ACSL1: Acyl-CoA synthetase long-chain family member 1
FADS1: Fatty acid desaturase 1
SCD5: Stearoyl-CoA desaturase 5
AGPAT2: 1-acylglycerol-3-phosphate O-acyltransferase 2
DGAT2: Diglyceride acyltransferase 2
APOA4: Apolipoprotein A4
SLC27A1: Solute carrier family 27 member 1

## References

[1] Emmerson DA (1997). Commercial approaches to genetic selection for growth and feed conversion in domestic poultry. Poult Sci.

[2] Geraert PA, MacLeod MG, Larbier M, Leclercq B (1990). Nitrogen metabolism in genetically fat and lean chickens. Poult Sci.

[3] Zhang XY, Wu MQ, Wang SZ, Zhang H, Du ZQ, Li YM (2018). Genetic selection on abdominal fat content alters the reproductive performance of broilers. Animal.

[4] Leclercq B, Blum JC, Boyer JP (1980). Selecting broilers for low or high abdominal fat: initial observations. Br Poult Sci.

[5] Leclercq B, Leclercq B, Whitehead CC (1988). Genetic selection of meat-type chickens for high or low abdominal fat content. Leanness in domestic birds: genetic, metabolic and hormonal aspects.

[6] Le Bihan-Duval E, Mignon-Grasteau S, Millet N, Beaumont C (1998). Genetic analysis of a selection experiment on increased body weight and breast muscle weight as well as on limited abdominal fat weight. Br Poult Sci.

[7] Ali AT, Hochfeld WE, Myburgh R, Pepper MS (2013). Adipocyte and adipogenesis. Eur J Cell Biol.

[8] Lefterova MI, Lazar MA (2009). New developments in adipogenesis. Trends Endocrinol Metab.

[9] Siersbaek R, Nielsen R, Mandrup S (2012). Transcriptional networks and chromatin remodeling controlling adipogenesis. Trends Endocrinol Metab.

[10] Fouad AM, El-Senousey HK (2014). Nutritional factors affecting abdominal fat deposition in poultry: a review. Asian Australas J Anim Sci.

[11] Lee JE, Schmidt H, Lai BB, Ge K (2019). Transcriptional and epigenomic regulation of adipogenesis. Mol Cell Biol.

[12] Abdalla BA, Chen J, Nie QH, Zhang XQ (2018). Genomic insights into the multiple factors controlling abdominal fat deposition in a chicken model. Front Genet.

[13] Bartel DP (2004). MicroRNAs: genomics, biogenesis, mechanism, and function. Cell.

[14] Victor A (2004). The functions of animal microRNAs. Nature.

[15] Price NL, Fernandez-Hernando C (2016). miRNA regulation of white and brown adipose tissue differentiation and function. Biochim Biophys Acta.

[16] Peng Y, Yu S, Li H, Xiang H, Peng J, Jiang S (2014). MicroRNAs: emerging roles in adipogenesis and obesity. Cell Signal.

[17] McGregor RA, Choi MS (2011). microRNAs in the regulation of adipogenesis and obesity. Curr Mol Med.

[18] Huang HY, Liu RR, Zhao GP, Li QH, Zheng MQ, Zhang JJ, Li SF, Liang Z, Wen J (2015). Integrated analysis of microRNA and mRNA expression profiles in abdominal adipose tissues in chickens. Sci Rep.

[19] Wang Z, Zhao QS, Li XQ, Yin ZT, Chen SR, Wu S, et al. MYOD1 inhibits avian adipocyte differentiation via miRNA-206/KLF4 axis. J Anim Sci Biotechnol. 2021;12:55. 10.1186/s40104-021-00579-x. 10.1186/s40104-021-00579-x PMC8101123 33952351

[20] Wang WS, Cheng M, Qiao SP, Wang YX, Li H, Wang N (2017). Gga-miR-21 inhibits chicken pre-adipocyte proliferation in part by down-regulating Kruppel-like factor 5. Poult Sci.

[21] Zhang XF, Song H, Qiao SP, Liu J, Xing TY, Yan XH, Li H, Wang N (2017). MiR-17-5p and miR-20a promote chicken cell proliferation at least in part by upregulation of c-Myc via MAP 3K2 targeting. Sci Rep.

[22] Wang W, Zhang TM, Wu CY, Wang SS, Wang YX, Li H, Wang N (2017). Immortalization of chicken preadipocytes by retroviral transduction of chicken TERT and TR. PLoS One.

[23] Tian WH, Zhang B, Zhong H, Nie RX, Ling Y, Zhang H, Wu C (2021). Dynamic expression and regulatory network of circular RNA for abdominal preadipocytes differentiation in chicken (Gallus gallus). Front Cell Dev Biol.

[24] Langmead B, Trapnell C, Pop M, Salzberg SL (2009). Ultrafast and memory-efficient alignment of short DNA sequences to the human genome. Genome Biol.

[25] Kozomara A, Birgaoanu M, Griffiths-Jones S (2019). miRBase: from microRNA sequences to function. Nucleic Acids Res.

[26] Love MI, Huber W, Anders S (2014). Moderated estimation of fold change and dispersion for RNA-seq data with DESeq2. Genome Biol.

[27] Betel D, Wilson M, Gabow A, Marks DS, Sander C (2008). The microRNA.org resource: targets and expression. Nucleic Acids Res.

[28] Lewis BP, Shih IH, Jones-Rhoades MW, Bartel DP, Burge CB (2003). Prediction of mammalian microRNA targets. Cell.

[29] Ernst J, Bar-Joseph Z (2006). STEM: a tool for the analysis of short time series gene expression data. BMC Bioinformatics.

[30] Kim D, Langmead B, Salzberg SL (2015). HISAT: a fast spliced aligner with low memory requirements. Nat Methods.

[31] Pertea M, Pertea GM, Antonescu CM, Chang TC, Mendell JT, Salzberg SL (2015). StringTie enables improved reconstruction of a transcriptome from RNA-seq reads. Nat Biotechnol.

[32] Yu G, Wang LG, Han Y, He QY (2012). clusterProfiler: an R package for comparing biological themes among gene clusters. OMICS.

[33] Hu H, Miao YR, Jia LH, Yu QY, Zhang Q, Guo AY (2019). AnimalTFDB 3.0: a comprehensive resource for annotation and prediction of animal transcription factors. Nucleic Acids Res.

[34] Grant CE, Bailey TL, Noble WS (2011). FIMO: scanning for occurrences of a given motif. Bioinformatics.

[35] Fornes O, Castro-Mondragon JA, Khan A, van der Lee R, Zhang X, Richmond PA, Modi BP, Correard S, Gheorghe M, Baranašić D, Santana-Garcia W, Tan G, Chèneby J, Ballester B, Parcy F, Sandelin A, Lenhard B, Wasserman WW, Mathelier A (2020). JASPAR 2020: update of the open-access database of transcription factor binding profiles. Nucleic Acids Res.

[36] Ye J, Coulouris G, Zaretskaya I, Cutcutache I, Rozen S, Madden TL (2012). Primer-BLAST: a tool to design target-specific primers for polymerase chain reaction. BMC Bioinformatics.

[37] Farmer SR (2006). Transcriptional control of adipocyte formation. Cell Metab.

[38] Ma YD, Zhang HY, He XL, Song HX, Qiang YY, Li Y, Gao J, Wang Z (2015). miR-106a*inhibits the proliferation of renal carcinoma cells by targeting IRS-2. Tumor Biol.

[39] Gong C, Qu S, Liu B, Pan S, Jiao Y, Nie Y, Su F, Liu Q, Song E (2015). MiR-106b expression determines the proliferation paradox of TGF-beta in breast cancer cells. Oncogene.

[40] Yao YL, Wu XY, Wu JH, Gu T, Chen L, Gu JH, Liu Y, Zhang QH (2013). Effects of microRNA-106 on proliferation of gastric cancer cell through regulating p21 and E2F5. Asian Pac J Cancer Prev.

[41] Shi DM, Bian XY, Qin CD, Wu WZ (2018). miR-106b-5p promotes stem cell-like properties of hepatocellular carcinoma cells by targeting PTEN via PI3K/Akt pathway. Onco Targets Ther.

[42] Li HL, Li TP, Wang SH, Wei JF, Fan JF, Li J, Han Q, Liao L, Shao C, Zhao RC (2013). miR-17-5p and miR-106a are involved in the balance between osteogenic and adipogenic differentiation of adipose-derived mesenchymal stem cells. Stem Cell Res.

[43] Pearson R, Fleetwood J, Eaton S, Crossley M, Bao S (2008). Krüppel-like transcription factors: a functional family. Int J Biochem Cell B.

[44] Gray S, Feinberg MW, Hull S, Kuo CT, Watanabe M, Sen-Banerjee S, DePina A, Haspel R, Jain MK (2002). The Kruppel-like factor KLF15 regulates the insulin-sensitive glucose transporter GLUT4. J Biol Chem.

[45] Kulyte A, Ehrlund A, Arner P, Dahlman I (2017). Global transcriptome profiling identifies KLF15 and SLC25A10 as modifiers of adipocytes insulin sensitivity in obese women. PLoS ONE.

[46] Matoba K, Lu Y, Zhang R, Chen ER, Sangwung P, Wang B, Prosdocimo DA, Jain MK (2017). Adipose KLF15 controls lipid handling to adapt to nutrient availability. Cell Rep.

[47] Mori T, Sakaue H, Iguchi H, Gomi H, Okada Y, Takashima Y, Nakamura K, Nakamura T, Yamauchi T, Kubota N, Kadowaki T, Matsuki Y, Ogawa W, Hiramatsu R, Kasuga M (2005). Role of Kruppel-like factor 15 (KLF15) in transcriptional regulation of adipogenesis. J Biol Chem.

[48] Antin PB, Pier M, Sesepasara T, Yatskievych TA, Darnell DK (2010). Embryonic expression of the chicken Kruppel-like (KLF) transcription factor gene family. Dev Dyn.

[49] Lyu SJ, Tian YD, Wang SH, Han RL, Mei XX, Kang XT (2014). A novel 2-bp indel within Kruppel-like factor 15 gene (KLF15) and its associations with chicken growth and carcass traits. Br Poult Sci.

[50] Wang YM, Xu YO, Wang ZM, Xu LY, Yang L, Lin YQ (2019). Studies on the cloning of KLF15 gene, tissue expression profile and the association between its expression and intramuscular fat content in Tibetan chicken. Chin J Anim Vet Sci.

[51] Matsubara Y, Aoki M, Endo T, Sato K (2013). Characterization of the expression profiles of adipogenesis-related factors, ZNF423, KLFs and FGF10, during preadipocyte differentiation and abdominal adipose tissue development in chickens. Comp Biochem Physiol B Biochem Mol Biol.

[52] Zhang M, Li F, Ma XF, Li WT, Jiang RR, Han RL, Li GX, Wang YB, Li ZY, Tian YD, Kang XT, Sun GR (2019). Identification of differentially expressed genes and pathways between intramuscular and abdominal fat-derived preadipocyte differentiation of chickens in vitro. BMC Genomics.

[53] Tontonoz P, Spiegelman BM (2008). Fat and beyond: the diverse biology of PPAR gamma. Annu Rev Biochem.

[54] Tunim S, Phasuk Y, Aggrey SE, Duangjinda M. Increasing fat deposition via upregulates the transcription of peroxisome proliferator-activated receptor Gamma in native crossbred chickens. Animals (Basel). 2021;11(1):90. 10.3390/ani11010090. 10.3390/ani11010090 PMC7824829 33466503

[55] Wang L, Na W, Wang YX, Wang YB, Wang N, Wang QG, et al. Characterization of chicken PPARγ expression and its impact on adipocyte proliferation and differentiation. Hereditas(Beijing). 2012;34(4):454–64. 10.3724/sp.j.1005.2012.00454. 10.3724/sp.j.1005.2012.00454 22522163

[56] Qiu FF, Xie L, Ma JE, Luo W, Zhang L, Chao Z (2017). Lower expression of SLC27A1 enhances intramuscular fat deposition in chicken via down-regulated fatty acid oxidation mediated by CPT1A. Front Physiol.

[57] Qi RL, Feng M, Tan X, Gan L, Yan GY, Sun C (2013). FATP1 silence inhibits the differentiation and induces the apoptosis in chicken preadipocytes. Mol Biol Rep.

[58] Zhao ZD, Tian HS, Shi BG, Jiang YY, Liu X, Hu J (2019). Transcriptional regulation of the bovine fatty acid transport protein 1 gene by kruppel-Like factors 15. Animals (Basel).

[59] Schmid B, Rippmann JF, Tadayyon M, Hamilton BS (2005). Inhibition of fatty acid synthase prevents preadipocyte differentiation. Biochem Biophys Res Commun.

[60] Zhao J, Sun XB, Ye F, Tian WX (2011). Suppression of fatty acid synthase, differentiation and lipid accumulation in adipocytes by curcumin. Mol Cell Biochem.

[61] Jung DY, Chalasani U, Pan N, Friedline RH, Prosdocimo DA, Nam M (2013). KLF15 is a molecular link between endoplasmic reticulum stress and insulin resistance. PLoS ONE.

